# Host and Phenology Shifts in the Evolution of the Social Moth Genus *Thaumetopoea*

**DOI:** 10.1371/journal.pone.0057192

**Published:** 2013-02-27

**Authors:** Mauro Simonato, Andrea Battisti, Carole Kerdelhué, Christian Burban, Carlos Lopez-Vaamonde, Isabelle Pivotto, Paola Salvato, Enrico Negrisolo

**Affiliations:** 1 DAFNAE-Entomology, University of Padua, Padua, Italy; 2 INRA, UMR CBGP (INRA/CIRAD/IRD/Montpellier Supagro), Montferrier-sur-Lez, France; 3 INRA, UMR1202 BIOGECO (INRA/Université de Bordeaux), Cestas, France; 4 INRA, UR033 URZF, Orléans, France; 5 BCA Department of Comparative Biomedicine and Food Safety, University of Padua, Padua, Italy; CNRS, Université de Bourgogne, France

## Abstract

The genus *Thaumetopoea* contains the processionary moths, a group of lepidopteran associated with forest trees, well known for the social behaviour of the larvae and for carrying urticating setae. The taxonomy of the genus is partly unresolved and a phylogenetic approach is lacking. The goal of this work is to produce a phylogeny for *Thaumetopoea* and to identify the main traits driving the evolution of this group. Eighteen mitochondrial and three nuclear genes were fully/partly sequenced. Markers were aligned and analysed singularly or in various combinations. Phylogenetic analyses were performed according to maximum likelihood and Bayesian inference methods. Trees obtained from largest data sets provided identical topologies that received strong statistical support. Three main clades were identified within *Thaumetopoea* and were further supported by several signatures located in the mitochondrial tRNAs and intergenic spacers. The reference topology was used to investigate the evolution of life history traits related to biogeography, host plant, ecology, and morphology. A multigenic approach allowed to produce a robust phylogenetic analysis of the genus *Thaumetopoea*, with the identification of three major clades linked to different ecological and life history traits. The first clade is associated with Angiosperm host plants and has a fast spring development of larvae on young foliage. The other clades have originated by one event of host plant shift to Gymnosperm Pinaceae, which implied a longer larval developmental time due to the lower nutritional quality of leaves. These clades showed different adaptations to such a constraint, the first with a switch of larval feeding to cold season (winter pine processionary moths), and the second with a retraction to high altitude and latitude and a development cycle extended over two years (summer pine processionary moths). Recent global warming is affecting all species and seems able to further shape the evolution of the group.

## Introduction

Evolution and diversification of animal species often result from a number of adaptive mechanisms to the environmental conditions [1]. This is particularly true for herbivorous insects, which are strongly dependent on both abiotic and biotic variables determining their fitness in different habitats. Major hurdles are represented by limitations imposed by temperature [2], by the availability of suitable plants [3] and by the complex of natural enemies associated with them [4]. A number of evolutionary strategies have been adopted to cope with such limitations and have resulted in a large diversity of adaptations, often leading to reproductive isolation and speciation.

Host plant diversity seems to be the main source of diversification in herbivorous insects [5], as host plant shifts (i.e. specialization onto a novel host) have been recorded in almost 50% of speciation events [6]. Host plant shifts and host plant range expansion, driven by climatic changes, can lead to further speciation events as they can increase the geographical range of insect species, creating opportunities for later geographical isolation or local adaptation to new environmental conditions [7]. Host shifts are often the result of a trade-off between host plant availability/quality and the natural enemies associated with this particular habitat [8].

Lepidoptera are, with very few exceptions, one of the best examples of evolution driven by host plants from one side [9], and by parasitoids and predators on the other side [10], [11]. Several characteristics in a host plant are relevant for larvae such as nutritional quality, toughness and presence of toxic allelochemicals. Lepidoptera have developed a variety of methods to circumvent plant defences that include both behavioural and biochemical/physiological adaptations [12]. Host shifts are thus expected to be more frequent between closely related host plants (same genus/family) [13]. On the other hand, similarities in secondary metabolic compounds might explain host shifts between unrelated plants [14], [15] such as in some butterfly species of the genus *Papilio* (Lepidoptera: Papilionidae) that shifted from Rutaceae to Apiaceae [16] or in the leaf-mining moth *Acrocercops leucophaea* complex (Lepidoptera: Gracillariidae), where two species were supposed to shift independently from Juglandaceae to Ericaceae [17].

Protection from both environmental conditions and natural enemies is considered to be one of the main causes for the social behavioural patterns found in the larval stage of several families of butterflies and moths [18]. Larvae may aggregate in early or later instars, either unprotected or protected by bark, leaf surfaces or silk tents built by the caterpillars, depending on their foraging patterns and their defence strategies [19]. In some species like the eastern tent caterpillar *Malacosoma americanum* (Lepidoptera: Lasiocampidae), larvae group together in a silk tent mostly to regulate their body temperature as they feed in periods of the year (such as winter or early spring) when temperatures are not optimal for a rapid development.

The genus *Thaumetopoea* Hübner (Lepidoptera: Notodontidae: Thaumetopoeinae) includes a dozen of species commonly known as processionary moths, occurring mainly in the Western Palaearctic region [20], [21], where a few species are serious pests in forestry [22], [23]. Most species are distributed on the southern range of this region (i.e. the Mediterranean Basin), but a few are found in more northern areas and eventually quickly expand to the north in response to climate change [24]. The larvae feed on trees and shrubs of resin-rich families such as Pinaceae (pine, cedar), Anacardiaceae (pistachio, sumac), and Cistaceae, with the exception of one species feeding on Fagaceae (oak) which lack resin. The larvae share a very peculiar defence system, consisting of urticating setae that are released upon attack of vertebrate predators, and are a nuisance to both humans and domestic animals [25].

The objective of the present study was to produce a multi-gene phylogeny of the genus *Thaumetopoea*, and to use it to reveal life history trait evolution, i.e. whether traits evolved several times or were rather stable within clades (phylogenetic conservatism). Furthermore, the phylogeny helped to clarify the taxonomy of the group, testing if the morphological characters used to delimit taxa were consistent with the phylogenetic relationships or rather convergent or adaptive. Since nuclear and mitochondrial genes have different inheritance patterns, sequences of mitochondrial genes combined with the sequences of nuclear genes can give a better insight of the phylogenetic relationships among different taxa [26]. To date most of the phylogenetic analyses including large parts of/complete mitochondrial genomes have been made using the protein-coding genes (e.g. [27]). Conversely, very few studies have included the tRNAs sequences (e.g. [28], [29]) and no-one has used information derived from secondary structure to identify molecular signatures at any taxonomic level. Analogously, even if the potential value of the mitochondrial intergenic spacers (isp) as phylogenetic/diagnostic markers has been postulated for Lepidoptera [30], no work has been performed so far to explore this possibility. Thus, an additional goal of this study was to test if tRNAs and isps provide molecular signatures that allow to define species boundaries and/or phylogenetic relationships among taxa.

## Materials and Methods

### Studied system and sampling

The genus *Thaumetopoea* Hübner *sensu lato* includes a dozen of species in the Palaearctic region, and one in the Afrotropical region [20], [21]. It has been split into two genera (*Thaumetopoea* Hübner *sensu stricto* and *Traumatocampa* Wallengren) by de Freina and Witt [31], with a further separation of the new genus *Heliantocampa* de Freina & Witt from *Traumatocampa* [32] using morphological traits of the adults. According to these authors, solely *Thaumetopoea processionea* (Linnaeus, 1758) and *Thaumetopoea solitaria* (Freyer, 1838) belong to the *Thaumetopoea* genus while all the other species belong to *Traumatocampa* (*T. apologetica* Strand, 1909; *T. bonjeani* (Powell, 1922); *T. cheela* Moore, 1883; *T. jordana* (Staudinger, 1894); *T. libanotica* Kiriakoff & Talhouk, 1975; *T. pinivora* (Treitschke, 1834); *T. pityocampa* (Denis and Schiffermüller, 1755); *T. wilkinsoni* Tams, 1925), with the only exception of one species assigned to the new genus *Heliantocampa* (*H. herculeana* (Rambur, 1840)). Another species was described from Arabic peninsula (Oman) as *Thaumetopoea dhofarensis* [33] and not included in the revision of de Freina and Witt [31]. According to Wiltshire [33], *T. dhofarensis* belongs to the *apologetica* group and thus fits into *Traumatocampa*. Furthermore, three new species were described later from Turkey and assigned to the *Traumatocampa* genus. They are *Traumatocampa ispartaensis* Doganlar & Avci, 2001, *Traumatocampa sedirica* Doganlar, 2005 and *Traumatocampa torosica* Doganlar, 2005 [34], [35]. Finally, new taxonomic issues arose from the genetic analysis of the most studied species (*T. pityocampa – T. wilkinsoni* group, [36], [37], [38]), indicating that the group actually consists of three species with a different geographic distribution. *T. pityocampa* is present in Europe and north-western Africa, *T. wilkinsoni* lives in the Middle East, and a new clade provisionally called *T. pityocampa* ENA (eastern-north Africa) occurs between Libya and eastern Algeria.

In the present work we initially sticked to the monophyletic view of the genus shared by Agenjo [20] and Kiriakoff [21] and then tested the split of de Freina and Witt [31], [32], by comparing a large set of molecular and biological data. We tried to get the most exhaustive coverage of the genus, although the limited knowledge on the geographic distribution of some taxa and the difficulties to access some countries did not allow to include the species from Africa (*T. apologetica*), India (*T. cheela*), Jordan (*T. jordana*), Oman (*T. dhofarensis*), and two of the three recently described species from Turkey (*T. sedirica* and *T. torosica*). The list of the *Thaumetopoea* species considered in the present work as well as the moth species used as outgroups is provided in Table 1, together with information on collection locality of specimens and the accession number of sequenced genes. One individual per species was used in the genetic analysis.

**Table 1:** List of the *Thaumetopoea* species and outgroups analysed in the present work, with indication of the collection data, and accession numbers of genes.

Family	Species	Site collection	Latitude	Longitude	Date	Legit	Accession numbers					
							*cox1*-*cox3*	*nad5*-*nad4*	*cob*-AT-rich region	*EF-1α*	*photolyase*	*wingless*
Arctiidae	*Hyphantria cunea*	MT, RN, LI	─	─	─	─	GU592049	GU592049	GU592049	U85671	─	EU333645
Lymantriidae	*Lymantria dispar*	MT, RN	─	─	─	─	FJ617240	FJ617240	FJ617240	U85672	─	EU333627
Notodontidae	*Ochrogaster lunifer*	Kenmore (Queensland), Australia	27° 30' S	152° 56' E	25/02/2005	M.P. Zalucki	AM946601	AM946601	AM946601	**HE861901**	**JX182499**	**HE861912**
Notodontidae	*Thaumetopoea bonjeani*	Forest of Chélia (Khenchela), Algeria	35° 22' N	6° 46'E	09/04/2006	M. Zamoum	**HE963107**	**HE864322**	**HE956692**	**HE861902**	**JX182487**	**HE861913**
Notodontidae	*Thaumetopoea herculeana*	Feces De Cima (Ourense), Spain	41° 51' N	7° 21′ E	04/04/2009	C. Lopez-Vaamonde	**HE963108**	**HE864323**	**HE956693**	**HE861903**	**JX182488**	**HE861914**
Notodontidae	*Thaumetopoea ispartaensis*	Kapidagi, Senirkent, Isparta, Turkey	37° 59’N	30° 36’ E	05/2006	M. Avci	**HE963109**	**HE864324**	**HE956694**	**HE861904**	**JX182489**	**HE861915**
Notodontidae	*Thaumetopoea libanotica*	Tannourine et Tahta, Lebanon	34° 12' N	35° 55' E	03/06/2006	N.Nemer, C.Lahousl	**HE963110**	**HE864325**	**HE956695**	**HE861905**	**JX182490**	**HE861916**
Notodontidae	*Thaumetopoea pinivora*	Gotland, Sweden	56° 18' N	18° 13' E	15/04/2006	A. Aimi	**HE963111**	**HE864326**	**HE956696**	**HE861907**	**JX182492**	**HE861917**
Notodontidae	*Thaumetopoea pityocampa*	Moggio (UD), Italy	46° 24' N	13° 12' E	12/03/2003	A. Battisti	**HE963112**	**HE864327**	**HE956697**	**HE861908**	**JX182493**	**HE861918**
Notodontidae	*Thaumetopoea pityocampa ENA (Eastern-Northern Africa)*	Bizerte, Tunisia	37° 02' N	9° 42' E	11/2000─	M. El Habib Ben Jamâa	**HE963113**	**HE864328**	**HE956698**	**HE861909**	**JX182497**	**HE861919**
Notodontidae	*Thaumetopoea processionea*	Caprino Veronese, Verona, Italy	45° 34' N	10° 46' E	21/5/2007	M. Faccoli, M. Zampini	**HE963114**	**HE864329**	**HE956699**	**HE861906**	**JX182495**	**HE861920**
Notodontidae	*Thaumetopoea solitaria*	Gamla (Golan heights), Israel	32° 59' N	35° 42' E	13/3/2005	A. Battisti	**HE963115**	**HE864330**	**HE956700**	**HE861910**	**JX182494**	**HE861921**
Notodontidae	*Thaumetopoea wilkinsoni*	Aladag, Turkey	37° 33' N	35° 22' E	28/3/2004	A. Battisti	**HE963116**	**HE864331**	**HE956701**	**HE861911**	**JX182496**	**HE861922**

─, not available; CR, mitochondrial control region. MT, [90]; RN, [91]; LI, [27]; bold underlined accession numbers, sequences produced for the present work.

The choice of outgroups was determined by two factors, i.e. their close relationships to the *Thaumetopoea* genus and the availability of sequences for most of the genes used in the present study. Three taxa were chosen as outgroups: *Ochrogaster lunifer,* the only species of Thaumetopoeinae (Lepidoptera: Notodontidae) with the full-length mitochondrial (mtDNA) genome already available [30]; *Lymantria dispar* (Linnaeus, 1758) (Lepidoptera: Erebidae: Lymantriinae) and *Hyphantria cunea* (Drury, 1770) (Lepidoptera: Erebidae: Arctiinae), that belong to the same superfamily, namely Noctuoidea [39]. Full length mtDNA genomes are available for both *L. dispar* (GenBank, unpublished) and *H. cunea* [27]. For these latter species, the sequences of two (*wingless*, *elongation factor EF-1α*) of the three nuclear genes analysed in this paper were also available (see below).

### Gene sampling

Three portions of the mtDNA genome were sequenced for each species of the *Thaumetopoea* genus. The first segment spanned from *cox1* to *cox3* (3481±268 bp), the second covered the *nad5*-*nad4* interval (2219±354 bp), the third had as boundaries *cob*-control region (*polyT*) (4185±220 bp). These three portions include the partial/complete sequence of eighteen mitochondrial genes. Three nuclear genes were also sequenced (see below for details). For the taxa used as out-groups, some of the data were obtained from GenBank, and new sequences were generated (see Table 1 for details).

The whole set of genes were chosen because it included fast and slow evolving markers obtained from both mitochondrial and nuclear genes, that exhibit different substitution rates, and represent a good mix for a multigenic approach aiming to solve phylogenetic/evolutionary issues.

### DNA extraction, PCR amplification and sequencing

Extraction of total DNA from ethanol-preserved larval specimens was performed either through a salting out-protocol [40] or using the GenElute mammalian Genomic DNA miniprep kit (Sigma). Quality of DNA was assessed through electrophoresis in a 1% agarose gel and staining with SYBR-safe DNA gel stain (Invitrogen).

Amplification and sequencing of partial/complete mitochondrial genes were performed using a mix of insect universal primers [41], [42], [43] and primers specifically designed against available sequences of different *Thaumetopoea* species (Table S1).

Amplification and sequencing of the nuclear genes *wingless* and *elongation factor EF-1α* were performed using universal primers [44]. PCR primers 5'-CCCTCCCCTATAATACATCC-3' and 5'-TTGAAAAGCCAAATTCATCT-3' were designed for the *photolyase* gene from cDNA libraries available for *T*. *pityocampa* (Kerdelhué, unpublished data).

All PCR products were directly sequenced using the primers used for amplification. In the case of *photolyase,* the sequencing was performed using the BigDye terminator v3 cycle sequencing kit (Applied Biosystems) and a ABI 3730 automatic sequencer at the Genomic and Sequencing Facility of Bordeaux. For all other genes the sequencing process was done by BMR Genomics (http://www.bmr-genomics.it/).

### Assembly of sequences and annotation

The mtDNA consensus sequences were assembled using the SeqMan II program from the Lasergene software package (DNAStar, Madison, WI). Gene and strand nomenclatures used in this paper follow Salvato et al. [30].

The mtDNA sequences were first translated *in silico* into putative proteins using the Transeq program, available at the EBI web site. The true identity of these polypeptides was established using the BLAST program available at the NCBI web site [45]. Gene boundaries were determined as follows. The 5’ ends of protein coding genes (PCGs) were inferred to be at the first legitimate in-frame start codon (ATN, GTG, TTG, GTT) in the open reading frame that was not located within the upstream gene encoded on the same strand [46], [47]. The only exception was *atp6* which overlaps with its upstream gene *atp8* in many mtDNAs (e.g. [30]).

The PCG terminus was inferred to be at the first in-frame stop codon encountered. When the stop codon was located within the sequence of a downstream gene encoded on the same strand, a truncated stop codon (T or TA) adjacent to the beginning of the downstream gene was designated the termination codon. This codon was thought to be completed by polyadenylation to a complete TAA stop codon after transcript processing. Finally, pair-wise comparisons with orthologous proteins were performed with ClustalW program to better define the limits of PCGs [48].

Irrespective of the real initiation codon, formyl-Met was assumed to be the starting amino acid for all proteins, as previously shown for other mitochondrial genomes [49], [50].

The transfer RNA genes were identified using the tRNAscan-SE program or recognized manually as sequences having the appropriate anticodon and capability of folding into the typical cloverleaf secondary structure [47], [51]. The identity of tRNAs was further corroborated by comparing them to their orthologous counterparts published for other lepidopteran species (e.g. [30]).

The boundaries of the ribosomal *rrnL* gene were assumed to be delimited by the ends of the *trnV*-*trnL1* pair. The 3’ end of the *rrnS* gene was assumed to be delimited by the start of *trnV*, while the 5’ end was determined through comparison with orthologous genes of other previously sequenced lepidopteran mtDNA genomes (e.g. [30]).

Assembly and annotation of *wingless* (*wng*), *elongation factor EF-1α* (*EF-1α*) and *photolyase* (*pho*) genes were straightforward. The assembly of the forward and reverse chromatograms was done through the SeqMan II program for *wng* and *EF-1α* and using CodonCode Aligner (www.codoncode.com) for *pho*. The identity of obtained consensus sequences was further corroborated through BLAST search, while the correctness of the frame was checked using the Transeq program.

### Sequence alignments

Calculation of pairwise distances and production of different file formats for phylogenetic purposes were done using MEGA5 program [52]. The multiple alignments (ALNs) of the genes (portion/full length) coding for proteins were produced using the pipeline implemented in the TranslatorX server [53]. This web-based tool allows to align orthologous nucleotide sequences using as backbone the alignment obtained for the corresponding translated polypeptides. The MAFFT program [54], [55], was used to align amino acid sequences in the TranslatorX server pipeline.

The ALNs of tRNAs, *rrnSs* and *rrnLs* were produced in two steps. Initially ALNs of orthologous sequences were created with the ClustalW program [48] followed by manual correction using as reference the secondary structures of tRNAs, *rrnSs* and *rrnLs*.

Reference structures for tRNAs were those identified by tRNAscan-SE program [51]. In the case of *rrnLs* and *rrnSs* new *ad hoc* secondary structures were produced. This process was done through an homology modelling approach, using as templates the published structures of *Drosophila melanogaster, Drosophila virilis, Libelloides macaronius* and *Manduca sexta* [56], [57], [58].

The mitochondrial genes sequenced in the present work included 7 tRNAs, the ribosomal *rrnL* and *rrnS*, and 9 protein encoding genes (mtPCGs). Nuclear genes *EF−1α* and *wng* were available for all species, conversely the *pho* gene was determined solely for Thaumetopoeinae. An ALN was created for every single gene/protein studied. The name of these ALNs are indicated below with the acronym used for the relative gene/protein.

ALNs covering various combinations of genes/proteins, as well as ALNs encompassing the whole set of genes/proteins, were analysed. These ALNs were produced through the concatenation of the ALNs obtained for single gene/protein.

Several multiple genes ALNs have self-explanatory names (e.g. *7trnas*). Less obvious ALNs are briefly described below. The *nuc2* (*EF−1α*+*wng*) and *nuc3* (*EF−1α*+*wng+pho*) ALNs differed for both genes and taxa composition. *Nuc2* contained all analysed taxa, while *nuc3* did not include *H. cunea* and *L. dispar,* because no *pho* sequences were available for these species. The *mtpcg* included the ALNs of the 9 mtPCGs. The *aga13sp*-*set* ALN was obtained through the concatenation of *mtpcg*, *nuc2*, *rrnL*, *rrnS* and *7trnas. The aag13sp*-*set* name refers to the fact it includes all (a) available (a) genes (partial/complete) (g) for the 13 species (sp) considered in this paper. The aga*11th*-*set* ALN was obtained from *aga13sp*-*set* by substituting *nuc2* with *nuc3* and by removing *H. cunea* and *L. dispar* species. Thus, the aag*11th*-*set* contains only moths belonging to Thaumetopoeinae (th). The amino acid ALNs encompassing more proteins were labelled with an acronym in capital letters identical to that of the nucleotide counterpart (e.g. NUC3 *vs*. *nuc3*).

### Phylogenetic analyses

An *a priori* estimation of the phylogenetic signal present in each ALN was performed by maximum likelihood mapping [59]. The phylogenetic signal was evaluated using the TREEPUZZLE 5.2 program [60]. The ALNs encompassing single tRNA did not exhibit enough phylogenetic signal, thus they were concatenated in a single alignment (7trnas, see above) for phylogenetic reconstruction.

Phylogenetic trees were inferred using Bayesian inference (BI) and maximum likelihood (ML) methods [61]. The BI trees were obtained with MrBayes 3.2 [62]. Two simultaneous runs, each of four chains, were performed in all analyses. Each run consisted of 1,000,000 generations, and trees were sampled every 100 generations. Stationarity was considered to be reached when the average standard deviation of split frequencies was less than 0.001. Burn-in was also increased respectively to 50%, 70%, and 90% without any appreciable change in tree topology and posterior probability values. Once stationarity was reached, a minimum of 1,000 trees was used to generate a majority-rule posterior consensus tree.

The ML analyses were performed with the RaxML 7.2.6 program [63] implemented in the graphical user interface raxmlGUI 0.93 [64]. The general time reversible substitution model (GTR) was applied to the ALNs containing protein-encoding genes [61] as well as to 7*trnas*, *rrnL* and *rrnS* alignments. For these latter data sets, we also applied the 16-state GTR model (S16) that takes into account the secondary structures [65]. As S16 model requires a secondary structure template to be applied, the secondary structures inferred for the seven tRNAs, *rrnL* and *rrnS* were used as template (see results). In the case of protein data sets the best fitting substitution matrix was identified comparing the likelihood scores obtained from trees built up using different matrices available in the RaxML program. Concerning the ALNs of the mitochondrial and nuclear encoded proteins (i.e. amino acids rather than DNA ALNs), the MTREV (mtDNA-encoded proteins reversible Markov model) and the JTT (Jones, Taylor, and Thornton) substitution matrices were respectively found as best fitting the data [66], [67]. In all ALNs the rate heterogeneity among sites was modelled using a four categories gamma distribution (G) without estimating the portion of invariable sites (I). Indeed the simultaneous estimation of G and I, even if very often applied, may provide statistically inconsistent results [68].

The effect of partitioning schemes were investigated in both BI and ML analyses. The number of data set partitions ranged from 1 to 15. Various partitioning schemes were applied to different ALNs to test their effects on the phylogenetic outputs. In the case of single protein encoding genes the first, second and third codon positions were considered as a single partition or treated independently in three distinct partitions. In multiple gene/protein ALNs a partition was applied to each gene/protein and further partitions were/were not applied to single codon positions.

Different partitioning schemes were applied also to *rrnS*, *rnnL*, *7trnas*, and to the ALNs obtained from combinations of these sets.

### Statistical tests on tree topologies

Posterior probabilities (PPs) were calculated for each node of the BI trees. Nonparametric bootstrap (BT) tests [69] were performed to assess the robustness of ML tree topologies (1,000 replicates in all cases). The approximately unbiased (AU) and the weighted Shimodaira and Hasegawa (WSH) tests [70] were performed to evaluate alternative phylogenetic hypotheses. ML scores necessary to calculate AU and WSH values were produced with the RaxML 7.2.6. program [63] and analysed with the Consel program [71]. Molecular dating was not possible due to the paucity of suitable calibration points.

The WSH test was used to assess the presence of incompatible phylogenetic signals among the single gene/protein ALNs. The reference tree obtained from *aag13sp*-*set* (see results) was evaluated against the best topology obtained for each single gene/protein ALN. Combinability of the single gene/protein ALNs in multiple genes/proteins data sets was considered possible if the *aag13sp*-*set* tree was not rejected by WSH test at p-value>0.01.

### Set of traits used to trace the evolutionary pathways of the *Thaumetopoea* genus

For each species, we compiled a list of traits grouped into four main categories, i.e. biogeography, host plant, ecology, and morphology. The list of the traits and their status is given in Supplementary material for all the species used for the present molecular study; they are also presented for the species that were not included in the molecular phylogeny whenever the information could be retrieved from the literature. The traits were selected based on their importance in the most studied species and on the use in the taxonomy of the group (Table S2).

Biogeographical traits were based on classical zoogeographic categories and on specific areas of occurrence, as far as reported in faunistic catalogues and in papers on species of applied importance (see references in Table S2). It was difficult to define the range of rare species. Individual reports of species occurrence were checked for consistency with the most recent taxonomic position of the studied taxa, and doubtful cases were excluded. The knowledge about the range of the host plants, in particular for those species with a widespread and continuous distribution over large areas, was useful to define the general distribution of taxa.

The host plants were derived from faunistic catalogues and specialized papers (see references in Table S2), although a careful scrutiny of the available information and cross-checking of the species had to be done to exclude cases of misidentification. These were mainly due to the oligophagous nature of many species, defined as the capacity to feed on more genera within a family of plants, within both Gymnosperms and Angiosperms. Mistakes were particularly evident in the last larval instars, when resources were depleted and the larvae could be found on non-host plant species. Records of this type were not included in the list of hosts. Host-plants were identified for all species known, with the exception of *T. apologetica* and *T. dhofarensis*, for which only adult stages are known. The presence/absence of resins (defined as hydrocarbon secretions of plants, produced and transported in specific resin ducts) [72] as defensive compounds was assessed on each host-plant genus. As resin is especially abundant in new leaves, the feeding of the larvae on young or mature foliage was also retrieved from life history papers, or deduced by the synchronization of the larval feeding with a specific phenological phase of the host plant.

As one prominent characteristic of the processionary moth is sociality at larval stage, four traits associated with this behaviour were identified and used in the analysis. First, foraging type was classified into three levels according to Fitzgerald [19], i.e. nomadic (larvae moving from one patch of food to another), patch-restricted (larvae living directly on their food source), and central place (fixed nesting site from which larvae move to get food). Second, the presence/absence of a conspicuous silk tent where the larvae rest when they are not feeding was recorded. Third, gregariousness as larva was classified as present throughout the whole stage or only in the early instars. Fourth, the pupation procession, consisting of head-to-tail line of larvae moving from the host plant to the pupation site, was recorded as presence/absence. Two more traits associated with life history and relevant for ecological adaptation were used. They are pupation site (silk tent, litter, soil) and overwintering stage (egg, larva, prepupa/pupa) of the typical univoltine cycle of all species of *Thaumetopoea*. The frequent possibility to enter prolonged diapause over one or more years was not considered because of lack of precise information in several species.

The morphological traits of adults, eggs, and larvae used for taxa identification were also classified for their presence/absence or status in the different species. This was the case for the front of the adults (crested or smooth) and for the occurrence of a prominent spine on the foreleg tibia (present/absent) [20], [31], [32]. The presence/absence of scales covering the egg clusters was evaluated as well as their shape, classified into combined categories of length (short: 0.7–0.8, medium: 1.4–1.9, long: 2.3–3 mm) and width (narrow: 0.2–0.5, medium: 0.7–0.9, wide 1.6–1.7 mm) [20]. The colour of the scale was also considered in previous taxonomic work but could not be used in the analysis because of lack of precise information in several species. The presence/absence of urticating setae on the larvae was evaluated based on the numerous reports summarised by Kiriakoff [21] and de Freina and Witt [32]. For the case of *T. herculeana*, reported to be non-urticating [20], the larvae used for the genetic analysis were inspected.

The traits were mapped on the reference phylogeny (see results) to trace their evolution within the *Thaumetopoea* genus. The ancestral character states were reconstructed by applying the parsimony algorithms implemented in McClade 4.08 program [73]. The analysed characters were treated as binary or multistate and as unordered [73].

## Results

### The mitochondrial tRNAs, rrnS and rrnL

**Figure 1:**
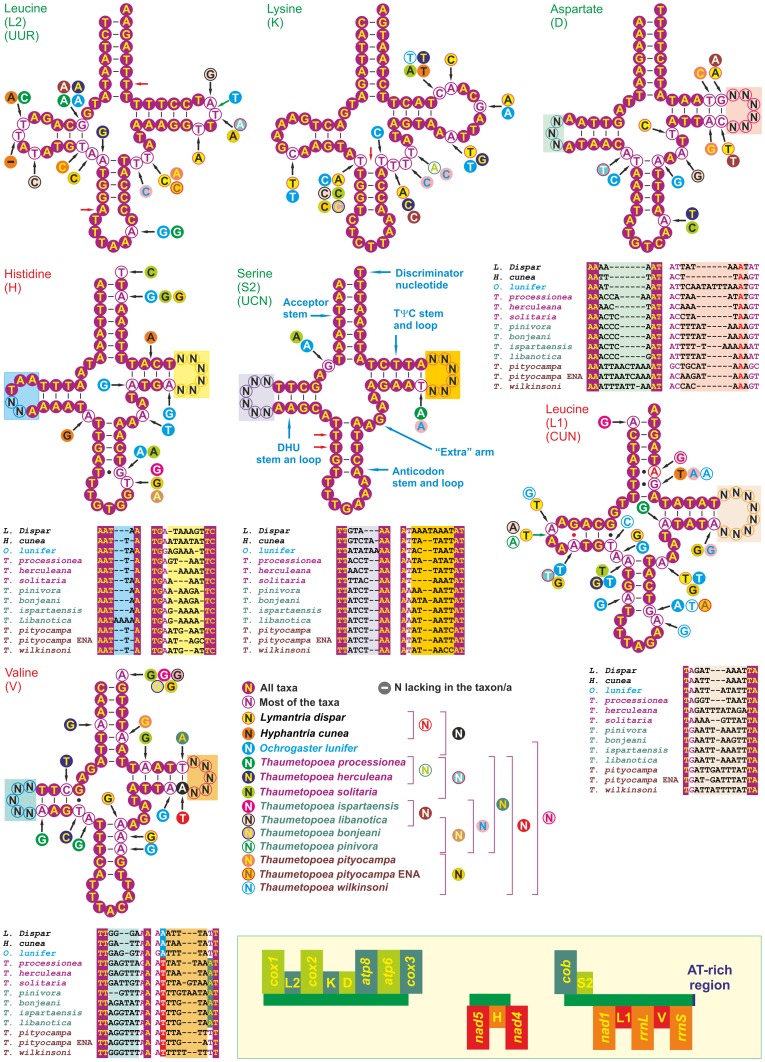
Secondary structure of and nucleotide substitution pattern in tRNAs. The nucleotide substitution pattern for each tRNA was modelled using as reference the consensus structure obtained from the structures of orthologous sequences. The substitution patterns for the very variable DHU and TΨC loops of some tRNAs are provided as portions of the corresponding complete multiple alignment immediately below the tRNA picture. Left bottom. The inset shows the positions of the sequenced tRNAs and their placement in the α (pale/dark green) or β strand (orange/red) of the typical lepidopteran complete mitochondrial genome.

The full-length sequences of seven tRNAs were determined and all of them exhibited the typical clover-leaf secondary structure (Fig. 1).

**Figure 2:**
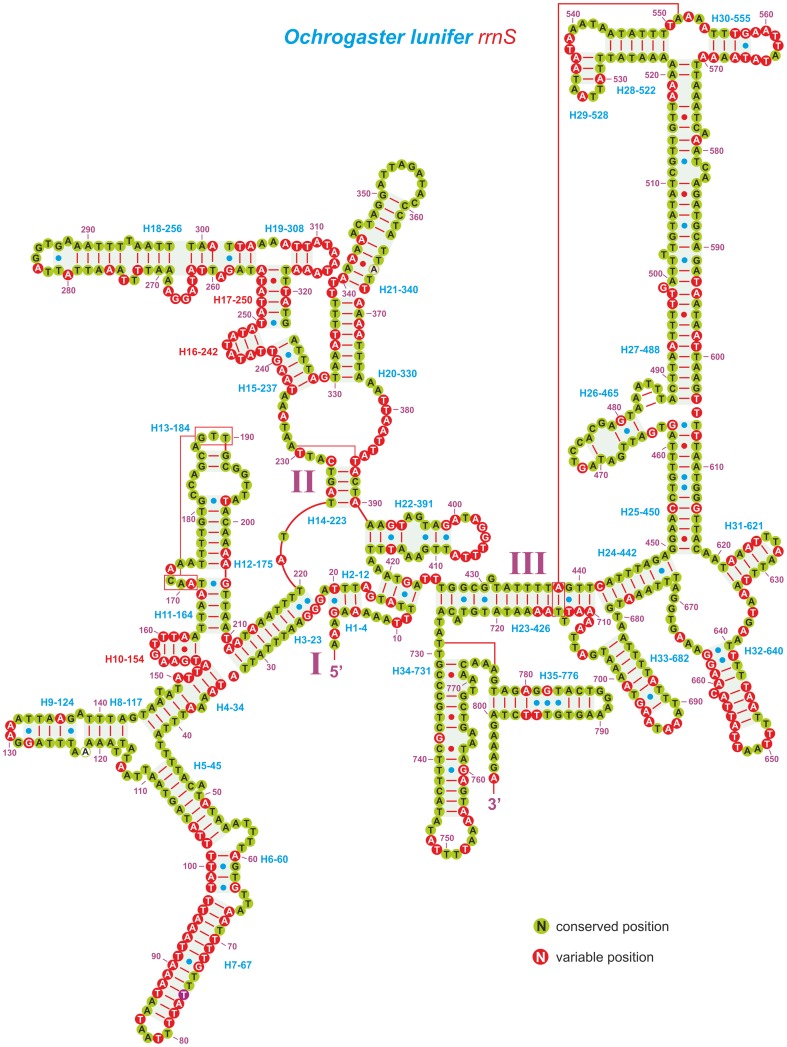
Secondary structure of *O*. *lunifer rrnS* and substitution patterns in the *rrnS* ALN. Each helix is numbered progressively from the 5’ to the 3’ end together with the first nucleotide belonging to the helix itself. Domains are labelled with Roman numerals. Tertiary structures are denoted by boxed bases joined by solid lines. Watson-Crick pairs are joined by dashes. GT pairs are joined by a blue dot, while other non-canonical pairs are connected by a red dot. Conserved position, position invariable in the *rrnS* ALN. Variable position, position not conserved in the *rrnS* ALN.

The secondary structure of the *rrnS* data set was modelled using as reference the sequence of *O*. *lunifer* (Fig. 2). This structure was mostly mirrored by other analysed species belonging to both in-group and out-groups.

**Figure 3:**
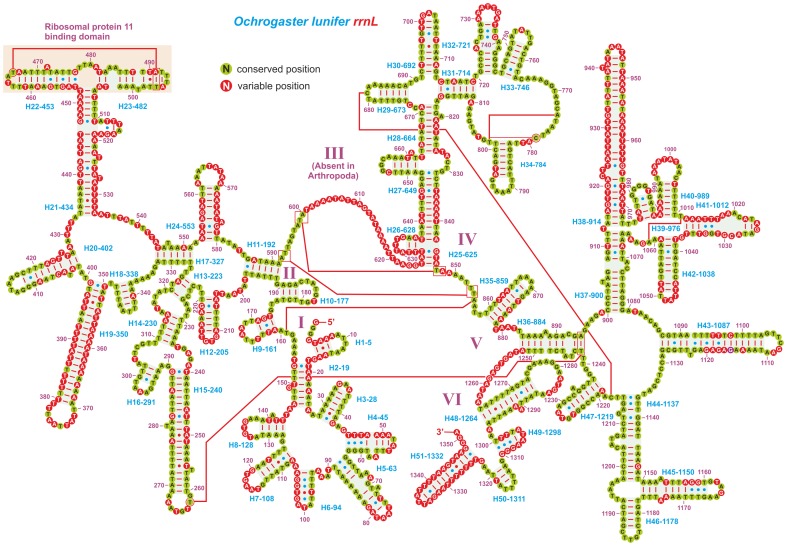
Secondary structure of *O*. *lunifer rrnL* and substitution patterns in the *rrnL* ALN. Each helix is numbered progressively from the 5’ to the 3’ end together with the first nucleotide belonging to the helix itself. Domains are labelled with Roman numerals. Tertiary structures are denoted by boxed bases joined by solid lines. Watson-Crick pairs are joined by dashes. GT pairs are joined by a blue dot, while other non-canonical pairs are connected by a red dot. Conserved position, position invariable in the *rrnL* ALN. Variable position, position not conserved in the *rrnL* ALN.

The secondary structure of the *rrnL* data set was also modelled using as reference the sequence of *O*. *lunifer* (Fig. 3). This structure was shared by all analysed species, even if the length/consistency of a few helices were different in various taxa. For all species it was possible to infer the structure of the domain I that it is usually left un-modelled (Fig. 3; Fig. S1).

### Phylogenetic signal detection

The phylogenetic signals detected in the different ALNs are summarized in Table 2. Third codon positions played a pivotal role in the phylogenetic signal of the protein-encoding genes ALNs (Table 2). Indeed they performed better than other single positions in terms of percentage of quartets fully resolved (%QFR). Furthermore their exclusion resulted in the drop of %QFR values that were very marked in some genes (e.g. *EF-1α*). Single tRNA exhibited very limited phylogenetic signal as proved by the low %QFR values and high percentages of quartets fully unresolved (%QFU). The ALNs obtained through concatenations of single gene/protein data sets exhibited the highest phylogenetic signals. This result was corroborated by the very high %QFR values and absence of %QFU.

**Table 2:** Name, length and phylogenetic signal of ALNs.

ALN name	S	length	%QFR	%QPU	%QFU	ALN name	S	length	%QFR	%QPU	%QFU
*atp8*	F	186	63.40	13.40	23.20	*nad5*	I	1,497	95.30	2.70	2.00
*atp8* p1-p2	F	124	60.60	6.80	32.60	*nad5* p1-p2	I	998	92.30	4.50	3.20
*atp8* p1	F	62	39.80	5.50	54.70	*nad5* p1	I	499	81.60	7.60	10.80
*atp8* p2	F	62	54.00	11.20	34.80	*nad5* p2	I	499	85.20	5.10	9.70
*atp8* p3	F	62	50.80	18.20	31.00	*nad5* p3	I	499	87.80	4.40	7.80
ATP8	F	62	62.70	6.80	30.50	NADH5	I	499	97.40	0.50	2.10
*atp6*	F	675	90.80	6.40	2.80	*rrnL*	F	1,400	96.20	2.00	1.80
*atp6* p1-p2	F	450	79.10	10.00	10.90	*rrnS*	F	787	97.00	2.20	0.80
*atp6* p1	F	225	67.60	9.90	22.50	*trnD*	F	78	38.90	10.80	50.30
*atp6* p2	F	225	70.10	7.20	22.70	*trnH*	F	71	57.00	10.00	33.00
*atp6* p3	F	225	82.30	5.70	12.00	*trnK*	F	71	36.00	19.70	44.30
ATP6	F	225	81.60	6.20	12.20	*trnL1*	F	72	55.10	11.60	33.30
*cob*	I	558	78.60	12.40	9.00	*trnL2*	F	68	54.50	5.90	39.60
*cob* p1-p2	I	372	62.70	13.50	23.80	*trnS2*	F	71	46.90	6.80	46.30
*cob* p1	I	186	63.20	11.10	25.70	*trnV*	F	71	51.10	6.40	42.50
*cob* p2	I	186	44.00	5.70	50.30	*EF-1α*	I	999	92.60	3.30	4.10
*cob* p3	I	186	69.10	7.00	23.90	*EF-1α* p1-p2	I	666	55.70	2.20	42.10
CYTB	I	186	67.30	6.50	26.2	*EF-1α* p1	I	333	52.60	4.70	42.70
*cox*1	I	1,458	93.40	4.40	2.20	*EF-1α* p2	I	333	0.00	0.00	100.00
*cox*1 p1-p2	I	972	77.50	12.20	10.30	*EF-1α* p3	I	333	90.60	4.50	4.90
*cox*1 p1	I	486	74.20	9.70	16.10	EF-1A	I	333	61.10	8.70	30.20
*cox*1 p2	I	486	57.10	7.50	35.40	*pho*	I	651	93.00	0.00	7.00
*cox*1 p3	I	486	87.30	4.30	8.40	*pho* p1-p2	I	434	81.50	2.40	16.10
COI	I	486	77.20	8.70	14.10	*pho* p1	I	217	75.50	0.00	24.50
*cox*2	F	681	86.30	7.30	6.40	*pho* p2	I	217	59.40	2.40	38.20
*cox*2 p1-p2	F	454	63.30	13.20	23.50	*pho* p3	I	217	86.40	1.20	12.40
*cox*2 p1	F	227	62.90	12.30	24.80	PHO	I	217	78.50	0.30	21.20
*cox*2 p2	F	227	56.90	5.60	37.30	*wng*	I	309	81.50	7.00	11.50
*cox*2 p3	F	227	82.90	8.00	9.10	*wng* p1-p2	I	206	54.70	2.50	42.80
COII	F	227	74.90	5.70	19.40	*wng* p1	I	103	48.00	2.50	49.50
*cox*3	I	102	52.00	11.20	36.80	*wng* p2	I	103	26.10	0.00	73.80
*cox*3 p1-p2	I	68	32.20	17.50	50.30	*wng* p3	I	103	76.60	6.80	16.60
*cox*3 p1	I	34	35.50	2.10	62.40	WNG	I	103	32.00	0.00	68.00
*cox*3 p2	I	34	39.00	21.10	39.60	nuc2	I	1,308	95.90	0.20	3.90
*cox*3 p3	I	34	42.90	5.40	51.70	*nuc2* p1-p2	I	872	67.50	7.70	24.80
COIII	I	34	26.80	10.80	62.40	*nuc2* p1	I	436	66.30	4.50	29.20
*nad1*	F	936	89.20	4.60	6.20	*nuc2* p2	I	436	26.10	0.00	73.80
*nad1* p1-p2	F	624	79.30	10.20	10.50	*nuc2* p3	I	436	92.30	3.60	4.10
*nad1* p1	F	312	67.10	9.50	23.40	NUC2	I	436	70.90	2.50	26.60
*nad1* p2	F	312	63.00	7.90	29.10	*nuc3*	I	1,959	96.30	0.10	3.60
*nad1* p3	F	312	79.80	6.80	13.40	*nuc3* p1-p2	I	1,306	86.30	1.00	12.70
NADH1	F	312	94.60	2.30	3.10	*nuc3* p1	I	653	86.40	0.90	12.70
*nad4*	I	267	63.10	7.40	29.50	*nuc3* p2	I	653	70.60	2.40	27.00
*nad4* p1-p2	I	178	57.40	9.00	33.60	*nuc3* p3	I	653	96.10	0.60	3.30
*nad4* p1	I	89	51.80	7.20	41.00	NUC3	I	653	88.80	0.00	11.20
*nad4* p2	I	89	41.40	3.60	55.00	mtpcg	I	6,360	99.20	0.20	0.60
*nad4* p3	I	89	54.90	9.00	36.10	*mtpcg* p1-p2	I	4,240	98.90	0.50	0.60
NADH4	I	89	63.40	4.70	31.90	*mtpcg* p1	I	2,120	97.40	0.80	1.80
*7trnas*	F	502	78.10	3.70	18.20	*mtpcg* p2	I	2,120	98.00	1.00	1.00
*rrnL+rrnS*	F	2,187	97.40	1.20	1.40	*mtpcg* p3	I	2,120	98.80	0.90	0.30
*rrnL+rrnS+7trnas*	F	2,689	99.00	1.00	0.40	mtPCG	I	2,120	99.60	0.40	0.00
*aag13sp*-*set*	I	10,357	99.60	0.40	0.00	*aag11th-set*	I	11,008	99.30	0.70	0.00
AAG13SP-SET	I	5,245	99.80	0.10	0.10	AAG11TH-SET	I	5,462	99.40	0.60	0.00

%QFR, percent of quartets fully resolved; %QPU, percent of quartets partly unresolved; %QFU, percent of quartets fully unresolved; F, ALN containing full-length gene/protein sequences; I, ALN containing incomplete gene/protein sequences; p1, p2 and p3, first, second and third positions of codons.

### Phylogenetic analyses

#### Tree topologies

ML trees were produced for the full-length ALNs listed in Table 2 (Fig. S2). This means that no trees were computed for protein-encoding data sets deprived of single/multiple codon positions. BI trees were produced solely for ALNs encompassing more than one gene/protein.

**Figure 4:**
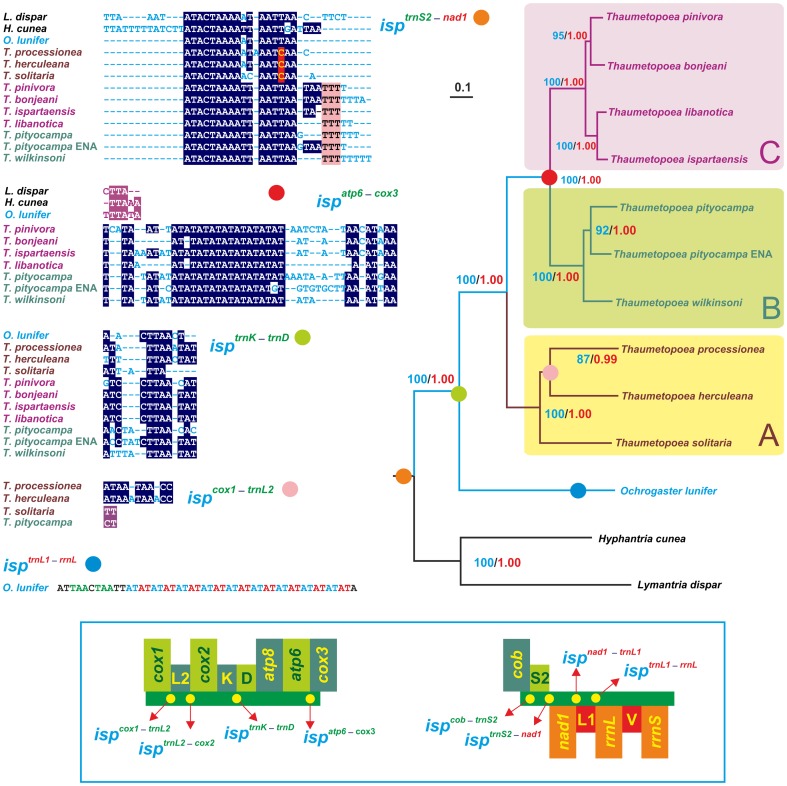
The reference phylogenetic tree and evolution of mitochondrial intergenic spacers. Maximum likelihood tree (−ln = 44305.147421) inferred from the *aag13sp*-*set* ALN. The analysis was performed by applying the GTR+G evolutionary model and according to the most complex partitioning scheme described in the main text. Blue-coloured numbers indicate bootstrap values expressed as percentage, whereas red-coloured numbers indicate posterior probabilities computed through Bayesian inference analysis on the same data set. The scale bar represents 0.1 substitutions/site. Occurrence and evolutionary pathway of isps exhibiting a uniqueness in term of genomic position plus sequence identity. Multiple alignments of isps, representing unique molecular signatures, are provided with invariant positions depicted on a blue background. Red/pink background is used to identify nucleotides characterizing peculiar clades. Single sequences/multiple alignments of isps, not representing unique molecular signatures, are depicted on a purple background. The inset on the bottom shows the placement of isps in the lepidopteran complete mitochondrial genome. Genes are coloured as in Figure 1.

The tree obtained from *aag13sp*-*set* is provided in Fig. 4, and represents our reference topology, because it was obtained from the data set exhibiting the maximum phylogenetic signal and including all the available taxa. Each node of the tree received strong/very strong PP (≥0.99) and BT (≥87%) supports. The *Thaumetopoea* species were split in three well supported clades named A, B, and C. Clade A included *T. herculeana*, *T. processionea* and *T. solitaria*. Clade B contained *T. pityocampa* ENA, *T. pityocampa* and *T. wilkinsoni*. The clade C encompassed *T. bonjeani*, *T. ispartaensis*, *T. libanotica,* and *T. pinivora*. Clades B and C were grouped as sister taxa with very high PP/BT support, while clade A represented an earlier branching off within the *Thaumetopoea* genus. Results were consistent whatever the phylogenetic method used (ML or BI, data not shown).

#### Impact of partitioning, secondary structures and outgroup choice

The different partitioning schemes applied to ALNs containing protein-encoding genes and/or polypeptides, did not alter the tree topology but affected the bootstrap of single nodes, which exhibited higher values when more elaborate partitioning strategies were applied.

The different evolutionary models applied to *rrnS*, *rnnL*, *7trnas*, and to the ALNs obtained from combinations of these sets, did not affect the obtained topologies that were consistent for each ALN. Thus exclusion/inclusion of secondary structure in the evolutionary model (GTR+G *vs.* S16) had not effect on the final tree. The application of single/multiple partitions did not change the tree topologies obtained from ribosomal and tRNA ALNs (i.e. *rrnL+rrnS* and *rrnL+rrnS+7trnas* data sets).

The two more external outgroups (i.e. *H*. *cunea* and *L*. *dispar*) were selectively removed from *aag13sp*-*set* ALN to test if this action would have affected the placement of the root for the *Thaumetopoea* genus. The trees obtained from this reduced sets were fully congruent with that presented in Fig. 4. This result proved that the placement of the ingroup root was not biased by the presence/absence of *H*. *cunea* and/or *L*. *dispar*. Phylogenetic analyses performed on *aag11th*-*set* and AAG11TH-SET ALNs, that contained the maximum number of analysed genes, but excluded *H*. *cunea* and *L*. *dispar*, resulted in trees having a topology perfectly matching the ingroup arrangement depicted in Fig. 4 (Figs. S2). These latter outcomes provided further evidence on the robustness of the subdivision of the *Thaumetopoea* genus into three major clades.

#### Tests of alternative topologies

The phylogenetic arrangement of *Thaumetopoea* species was further checked by testing alternative tree topologies. Calculations were performed on *aag13sp*-*set* and aag*11th*-*set* ALNs. Firstly the three major clades were disrupted by moving selectively one species to a different clade (e.g. *T. herculeana* placed as sister species of B+C). Then, different sister taxon relationships among A, B and C clades were tested (e.g. creation of a group A+C). All these alternative phylogenetic hypotheses were rejected by the AU test (P<0.001). These results further corroborated the three major clades and their phylogenetic relationships.

Situation within single clades was more variable. In the clade A all possible alternative groupings among the three species (e.g. *T. processionea*+*T. solitaria*) were not rejected by the AU test (P>0.05). Conversely, the clades B and C exhibited more fixed relationships and alternative arrangements among the species were rejected (P<0.05).

The presence of incompatible phylogenetic signals among single gene/protein ALNs was assessed through the WSH test. The reference topology of Fig. 4 was evaluated against the best topology obtained for each single gene/protein ALN. None of these data sets rejected the reference topology exhibiting p-values >/>> 0.01. These findings showed that the reference tree was a sub-optimal/optimal topology shared by all data sets thus corroborating the combinability of single gene/protein ALNs.

#### Within and among clades distances

Taking into account the obtained phylogenetic relationships, pairwise distances (p-distances) were calculated between all pairs of Thaumetopoeinae species for the *mtpcg* and *nuc3* data sets (Table 3). In the *mtpcg* ALN, *T. pityocampa* ENA *vs*. *T. pityocampa* exhibited a p-distance (0.074) higher than values observed for the pairs *T. bonjeani vs. T. pinivora* (0.047), and *T. ispartaensis vs*. *T. libanotica* (0.042). Analogously, in the *nuc3* ALN the p-distance of *T. pityocampa vs*. *T. pityocampa* ENA was 0.005, while the p-distance of *T. bonjeani vs. T. pinivora* was 0.003, and the p-distance of *T. ispartaensis vs*. *T. libanotica* was 0.002. Both ALNs, based exclusively on mitochondrial (*mtpcg*) and nuclear (*nuc3*) genes, corroborated a higher genetic differentiation between *T. pityocampa* and *T. pityocampa* ENA than those observed for the *T. bonjeani vs. T. pinivora* and *T. ispartaensis vs*. *T. libanotica* pairs.

**Table 3:** Pairwise p-distances between pairs of Thaumetopoeinae species.

	*O. lunifer*	*T. solitaria*	*T. herculeana*	*T. processionea*	*T. wilkinsoni*	*T. pityocampa* ENA	*T. pityocampa*	*T. ispartaensis*	*T. libanotica*	*T. bonjeani*	*T. pinivora*
	***mtpcg***										
*O. lunifer*	—										
*T. solitaria*	0.158	—									
*T. herculeana*	0.166	0.116	—								
*T. processionea*	0.169	0.124	0.125	—							
*T. wilkinsoni*	0.173	0.134	0.144	0.145	—						
*T. pityocampa* ENA	0.170	0.138	0.141	0.146	0.082	—					
*T. pityocampa*	0.168	0.138	0.142	0.142	0.083	0.074	—				
*T. ispartaensis*	0.175	0.138	0.138	0.144	0.117	0.114	0.122	—			
*T. libanotica*	0.170	0.134	0.135	0.140	0.113	0.112	0.116	0.042	—		
*T. bonjeani*	0.163	0.127	0.133	0.132	0.109	0.110	0.112	0.066	0.065	—	
*T. pinivora*	0.164	0.129	0.132	0.135	0.111	0.111	0.112	0.064	0.062	0.047	—
	***nuc3***										
*O. lunifer*	—										
*T. solitaria*	0.115	—									
*T. herculeana*	0.114	0.039	—								
*T. processionea*	0.113	0.031	0.033	—							
*T. wilkinsoni*	0.113	0.059	0.065	0.061	—						
*T. pityocampa* ENA	0.112	0.057	0.063	0.059	0.005	—					
*T. pityocampa*	0.112	0.056	0.062	0.058	0.006	0.005	—				
*T. ispartaensis*	0.116	0.061	0.067	0.060	0.031	0.030	0.029	—			
*T. libanotica*	0.115	0.061	0.066	0.060	0.032	0.031	0.029	0.002	—		
*T. bonjeani*	0.117	0.060	0.064	0.059	0.032	0.031	0.029	0.005	0.004	—	
*T. pinivora*	0.116	0.060	0.064	0.059	0.031	0.030	0.028	0.005	0.004	0.003	—

The *mtpcg* p-distances among clades were 0.142 for clade A *vs*. clade B; 0.135 for clade A *vs*. clade C; 0.114 for clade B *vs*. clade C. The average p-distances for *mtpcg* within each clade were: 0.122±0.003 for clade A; 0.080±0.002 for clade B; 0.058±0.002 for clade C. The *nuc3* p-distances among clades were: 0.060 for clade A *vs*. clade B; 0.062 for clade A *vs*. clade C; 0.030 for clade B *vs*. clade C. The average p-distances for *nuc3* within each clade were: 0.034±0.004 for clade A; 0.005±0.001 for clade B; 0.004±0.001 for clade C.

#### Mitochondrial signatures

Some molecular signatures, characterizing the major *Thaumetopoea* clades identified in the phylogenetic analysis (Fig. 4), were located in the DHU and TΨC loops of tRNAs (Fig. 1). For example, the DHU loop of *trnD* presented an AT-rich motif found only in clade A (brown-coloured *Thaumetopoea* species in Fig. 1) and a C-rich motif common to most species of clade B+C (green/purple coloured *Thaumetopoea* species in Fig. 1). Analogously, an AT-rich motif, exclusive of clade C, was located in TΨC loop of *trnD.* More motifs peculiar to different clades/species can be observed in Fig. 1 in the most divergent DHU and TΨC loops. In particular, *T. pityocampa* and *T. pityocampa* ENA exhibited distinct *trnH* TΨC loops (ATGAAT *vs*. ATAGC) in contrast with the common patterns observed in the closely related pairs *T. ispartaensis vs*. *T. libanotica* (GAAA) and *T. bonjeani vs. T. pinivora* (AAAGA). Further motifs that allowed to set apart *T. pityocampa* from *T. pityocampa* ENA were located on the DHU and TΨC loops of *trnD* and in the TΨC loop of *trnV*.

Three portions of the mitochondrial genome of *Thaumetopoea* species were sequenced in the present work. Two of them presented intergenic spacers (isps). The distribution and evolution of these spacers were mapped on the reference tree (Fig. 4). Three major patterns emerged. (1) The considered isp was common to all analysed species. This behaviour was observed for isp*^trnS2−nad1^*, that exhibited a rather conserved motif. Even within this homogenous isp some positions presented peculiar nucleotides characterizing clade A, and clade B+C (Fig. 4). (2) The isp was restricted to and characterized clades encompassing different numbers of species. This pattern was observed for isp*^trnK−trnD^*, isp*^atp6−cox3^* and isp*^cox1−trnL2^*. The isp*^trnK−trnD^* characterized all species belonging to Thaumetopoeinae (i.e. *O*. *lunifer* and *Thaumetopoea* species) but was absent in *H*. *cunea* and *L*. *dispar.* The isp*^atp6−cox3^* was present in the outgroups and in the B+C *Thaumetopoea* clade. However, when the sequence of this spacer was taken into account, it emerged that isp*^atp6−cox3^* of species belonging to B+C clade shared an exclusive long motif totally absent in the outgroups. The isp*^cox1−trnL2^* was present in four species of *Thaumetopoea* (Fig. 4). Again when the sequences were compared a common motif proved to be exclusive of the clade *T. herculeana*+*T. processionea*. The isp*^trnL1−rrnL^* was restricted to *O*. *lunifer*. In this case the isp represented a molecular signature exclusive of this species.

Finally, a further evidence of distinctiveness between *T. pityocampa* and *T. pityocampa* ENA was provided by the isp*^cox1−trnL2^*. In *T. pityocampa* the *cox1* gene ended with a complete TAA stop codon separated from the downstream *trnL2* gene by the isp*^cox1−trnL2^* made of the dinucleotide CT. Conversely in *T. pityocampa* ENA the *cox1* exhibited an incomplete T(aa) stop codon and was immediately adjacent to *trnL2* without an intervening isp*^cox1−trnL2^*.

### Evolutionary patterns of selected characters

**Figure 5:**
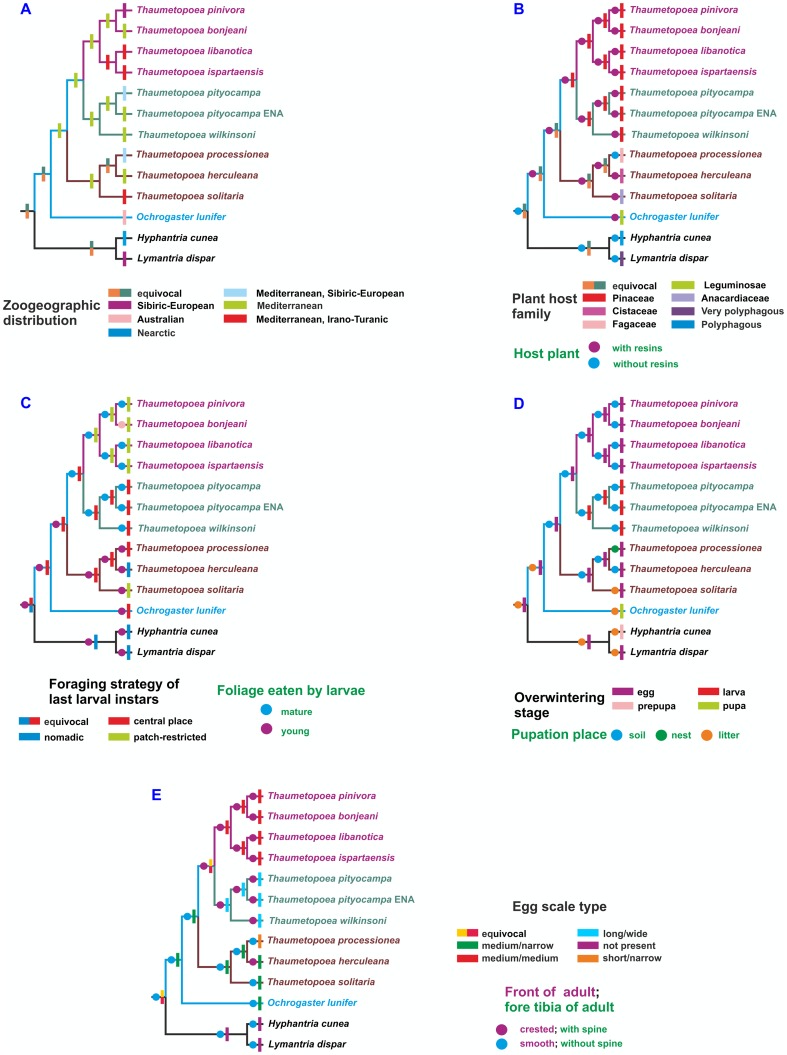
Evolution of selected characters in the *Thaumetopoea* genus. The evolutionary pathways of morphological, behavioural, and life history traits were reconstructed on the reference tree by applying the parsimony algorithms implemented in the McClade program.

The evolution of several traits, ranging from biogeography, host plants, ecology, and morphology was traced on the reference tree. This approach allowed to identify some of the features that characterized the common ancestor of *Thaumetopoea* moths or represented novelties restricted to some species of this genus (Fig. 5). The most interesting results of this analysis are described below and presented in the order they are displayed in Figs. 5A–5E.

The analysis of the biogeographic traits (Fig. 5A) indicated that the ancestor of the genus had a Mediterranean distribution, with at least one species with such a distribution in each of the three clades of *Thaumetopoea*. Successively, a shift to regions with continental climate (higher latitudes in Europe) occurred independently in each clade for *T. pinivora*, *T*. *processionea*, and *T. pityocampa*, respectively. A further differentiation in the Irano-Turanic area occurred for *T. libanotica* and *T. ispartaensis* in the clade C, and in *T. solitaria* in the clade A.

The analysis of the host plants shows that the genus *Thaumetopoea* is associated with four plant families, one in the Gymnosperms (Pinaceae) and three in the Angiosperms (Anacardiaceae, Cistaceae, Fagaceae) (Fig. 5B). All species feeding on Pinaceae belong to clades B and C, while all species feeding on Angiosperms are grouped in clade A. Species in clades A and B appear to be oligophagous, being able to feed on more than one genus in each host plant family, while specialization toward monophagy is observed in clade C, with species associated with *Pinus* (*T. pinivora*) or *Cedrus* (*T. bonjeani*, *T. ispartaensis*, *T. libanotica*). Presence of resin canals is common to all the host plants of *Thaumetopoea*, with the only exception of *T. processionea* which feeds on Fagaceae (mainly *Quercus* spp.). Larvae preferably feed on mature leaves on Gymnosperms (clades B and C) and on young leaves on Angiosperms (clade A).

The central place type foraging strategy seems to be an ancestral trait that characterized the *Thaumetopoea* species (Fig. 5C). This status was reverted to a patch-restricted foraging strategy, which emerged successively and independently in clade C and in *T. solitaria*. A further shift to the nomadic foraging strategy occurred in clade A for *T. herculeana*. Central place species are always associated with a large tent, which may be used for several months in species overwintering as an active larva (clade B) (Fig. 5C); this latter condition resulted to be an evolutionary novelty characterizing the clade B. In the other species only temporary tents are produced at the time of moulting, or no tent is produced in species loosing gregariousness in later instars (*T. herculeana*). Colony movement in head-to-tail lines is a typical feature of all larval instars during foraging bouts on the host plant, and it occurs for all species when searching for pupation sites in litter/soil away from the tree, with the only exception of *T. processionea* which pupates in the tent (Fig. 5D).

The pupation habit appears to be linked with major morphological traits of adult moths, as those pupating in the soil have crested front and a prominent spine on the foreleg tibia (Fig. 5E). These traits do not seem to be the ancestral trait of the group (Fig. 5E). According to the transformation pathway shown in the tree, both characters represent derived traits, that co-evolved independently twice within the genus (clade B+C *vs. T. herculeana*). Species that have switched to larval overwintering (clade B) show larger egg scales than those overwintering as an egg, which seems to be the ancestral trait (Fig. 5D and Fig. 5E). All larvae of *Thaumetopoea* carry urticating setae on the abdominal tergites starting from the third larval instar, including those of *T. herculeana*.

## Discussion

### Mitochondrial ribosomal and transfer RNAs

The secondary structures inferred for ribosomal and transfer RNA sequences analysed in the present paper largely agree with those previously determined for the Lepidoptera and more generally for the Insecta (e.g. [30], [56], [57], [58]).

The DHU and TΨC loops of different tRNAs present motifs that allow to discriminate among closely related taxa (e.g. *T. pityocampa* and *T. pityocampa* ENA) as well as to define phyletic lines within the genus *Thaumetopoea*. A dense taxon sampling is necessary to fully appreciate the potentials of these loops, as source of molecular signatures for closely related taxa. We expect that with the increasing availability of complete mtDNA genomes DHU and TΨC loops will provide a new class of molecular signature for phylogenetic/taxonomic purposes particularly in speciose genera.

It is possible to predict very accurately the secondary structures of tRNAs (e.g. [51]) and fairly well those of *rrnS* and *rrnL* genes (e.g. [30], [56], [57], [58]). This structural information should be, whenever possible, implemented in phylogenetic studies because it improves the quality of the multiple alignments and as a consequence that of obtained topologies (see [65]). Furthermore, the knowledge of the precise distribution of the molecular signatures along the secondary (tertiary) structure allows to identify, in a more biologically realistic context, what parts of the RNA gene support (at different taxonomic levels) the clades identified in the phylogenetic study.

### The intergenic mitochondrial spacers

Intergenic spacers are recursively found in the mitochondrial genomes of Lepidoptera (e.g. [30], [56]). A possible role of isps as molecular signature to define/characterize clades had previously been postulated for Lepidoptera (e.g. [30]). To our knowledge, the analysis presented in this work proves, for the first time, that this is the case.

The study of the distribution of isps along the tree branches of Fig. 4 evidenced that these genomic elements appeared recursively during the evolution and speciation of the species analysed in the present work. There is a fixed number of positions where isps may occur in the mitochondrial genome; consequently, a given isp can eventually be found at the same position in distant taxa (e.g. isp*^atp6−cox3^*, Fig. 4). However, when not only the position but also the sequence of each isp is taken into account and an alignment is produced, it appears that most isps represent strong molecular signatures characterizing monophyletic groups, that emerged in different moments of the cladogenetic process. This result shows that isps are an important class of mitochondrial signatures, that have been mostly neglected so far.

### Phylogeny and systematics of the *Thaumetopoea* genus

The agreement between phylogeny and the most recent taxonomic revision of the former genus *Thaumetopoea*, resulting in three genera [31], [32], is high but not complete. If the genus *Traumatocampa* matches perfectly with clades B and C, the winter and summer processionary moths feeding on Pinaceae, respectively, the other two genera *Thaumetopoea* and *Heliantocampa* are intermixed within clade A. Any attempt to remove *Heliantocampa* (i.e. *T. herculeana*) from clade A was rejected by the AU test. Conversely, the relative positions of the species within this clade can be interchanged without AU rejection, even if the *T. processionea*+*T. herculeana* relationship is robustly favoured by the reference topology as well as by molecular signatures against the alternative arrangements.

Based on a good correspondence between host plant taxonomy and the placement of the species in the clades, we may propose hypotheses about the position of taxa that for various reasons were not included in the analysis. Indeed all taxa feeding on Gymnosperms (Pinaceae) form the monophyletic group B+C, while the species associated with Angiosperms are included in clade A. Thus we hypothesize that *T. jordana* and *T. cheela*, both feeding on Anacardiaceae, belong to clade A, as they share the same host plant family with *T. solitaria* [22], [74]. The same could be proposed for *T. dhofarensis*, found in a habitat similar to that of *T. jordana* [33] and for *T. apologetica*, restricted to Eastern Africa where there are no native Pinaceae. Conversely, the two recently described species (*T. sedirica* and *T. torosica*, that feed on Pinaceae) are predicted to belong to either clade B or clade C.

Our molecular phylogenies strongly support a parallel evolution of the morphological traits used to divide *Thaumetopoea* in the three distinct genera *Traumatocampa*, *Heliantocampa* and *Thaumetopoea sensu stricto*. This result leads us to suggest that all species (included or not in the present work) should be treated as members of a single genus *Thaumetopoea sensu lato*. Further systematic revision should be delayed until a complete taxonomic coverage becomes available and the phylogenetic relationships among all species are fully resolved.

There is also a need for identifying new morphological synapomorphies that support the three identified clades, as those characters more commonly used (presence of the crest on the front of the adult and of the spine on the foreleg tibia, size of the egg scales) show evidence of convergent evolution. Indeed those characters are more related to ecological functions such as the emergence from the soil [75] and egg thermoregulation [76], and they are present in every clade (Table S2).

Genetic comparisons presented in this study strongly corroborate results initially obtained by Kerdelhué et al. [38] and favour the status of distinct species for *T. pityocampa* ENA, which should be separated from *T. pityocampa*. The distinctiveness between the two taxa is corroborated independently by protein-coding genes, both nuclear and mitochondrial, as well as mitochondrial signatures in tRNA loops. The clear-cut genetic differences are not mirrored by evident morphological features, and further studies will be necessary to explore the morphological and genetic variation as well as the geographic range of this taxon in North Africa.

### Evolution of traits in the *Thaumetopoea* genus

The tracing of the evolutionary pathway of each character identified a number of features that likely represent the ancestral condition for the genus. The ancestor of *Thaumetopoea* had a biogeographic distribution centred in the Mediterranean basin, and it probably derived from a stock of taxa living in the African continent where several Thaumetopoeinae genera occur, including one species of *Thaumetopoea* (*T. apologetica*) [21]. Geography does not seem to explain much of the present structure of the clades as species with similar distribution occur in each of the clades (for example, *T. processionea*, *T. pityocampa*, and *T. pinivora*). However we cannot exclude that current sympatric ranges are secondary. Furthermore, the spread outside the Mediterranean basin for *T. processionea, T. pityocampa*, and *T. pinivora* was probably linked to the range expansion of their host plants after the last Quaternary glaciation, and occurred independently in each clade. In addition, the species of clade B have responded to the recent climate change by a rapid expansion to higher altitude and latitude [24], [38], [77], while potential climatic-driven range shifts expected in clades A and C have been hypothesized [78], [79].

Shifts in host plant use seem indeed to be the main factor that determined speciation within the genus. The last common ancestor of *Thaumetopoea* moths exploited Angiosperm plants as larval food, but current evidence does not allow to unambiguously identify the exact family. The lack of specialization suggests that the common ancestor had the capability to cope with a broad array of host plant quality, and this could have been important in the splitting and radiation of the *Thaumetopoea* moths. The shift to Gymnosperms occurred just once and it may have been facilitated by similar characteristics in morphology (for example, foliage toughness) and phytochemistry (resins) of these plants with the ancestral broad-leaved host plants. Moreover, ancestral and new host plants belong to the same ecological association of plants adapted to poor soils and first colonizers of disturbed areas. The shift from broadleaf host plants to Pinaceae was associated with a dramatic loss of nutritional power, as pine needles contain at least three times less nitrogen than Angiosperm leaves [72], and this in turn implied a longer feeding and developmental time as a larva in order to compensate for the lower food quality. Thus the developmental time of the ancestor lineage, typically taking 2–3 months in spring, had to extend into summer, incurring in the high temperature of the Mediterranean region that can be fatal to larvae [80]. There are two ways by which the species associated to Pinaceae have responded to this strong constraint. The first is the change to the winter feeding observed in clade B, and the second is the retraction of the range to upper altitude and latitude observed in clade C. Both adaptations have a cost consisting in a longer exposure to natural enemies, and in protection from low temperature for species of clade B (tent construction, adoption of a central place foraging strategy).

In addition, the shift to winter feeding in clade B may have contributed to reduce the competition with other pine defoliating insects, especially sawflies, which are generally active in spring. As a further matter of facts, the reversion from winter to summer feeding recently observed in a population of coastal Portugal of *T. pityocampa*, which evolved a higher tolerance of the larvae to high temperature [80], indicates that the trait is still under selection and likely driven by climatic factors. The shift to winter feeding could also explain the larger egg scales of clade B, as they are functional to achieve an earlier hatching by increasing the egg temperature by several degrees [76].

The species of clade B are those reaching the higher size in the genus, followed by those of clade C and by the moths of clade A [20], [38]. Larvae of both B and C clades grow at lower temperatures than those of A lineage. Furthermore they exhibit a longer developmental time that is positively correlated with a larger body size even if in a complex way [81]. These observations would suggest some influence of the temperature on the size variation observed in the *Thaumetopoea* species. However the phenotype of a moth is the result of interactions amongst many genes and environmental factors [81]. The phenotypic variation can be determined largely/completely by genes or be heavily influenced by environmental factors acting epigenetically on the developmental genetic machinery [82]. Only carefully designed experiments allow to understand the contribution of single gene/environmental factor to the production of a particular phenotype [82]. Thus new studies are necessary to test if the temperature exerts a role on the size variation of different processionary moths.

The shift from Angiosperms to Gymnosperms corresponded also to a change of feeding from young to mature leaves. Such a change has been possible because Pinaceae retain leaves for three or more years but also because the mature leaves contain less defensive resin compounds than young leaves [72]. In addition, mature needles contain slightly more nitrogen than young needles [83]. It is interesting to observe that in the species of clade C the preference for mature needles has been maintained in spite of a spring development of the larvae [78], exactly when the young needles become available, indicating the avoidance of leaves with higher resin content.

Within Pinaceae, the species of clade B maintain a certain degree of polyphagy, being able to exploit host plants in different genera [84], [85], while in the clade C there is a specialization for either *Pinus* (*T. pinivora*) or *Cedrus* (the other three species). The shift on *Cedrus* could not be established before the Miocene, provided that this plant genus was not present in the Mediterranean basin before [86]. Here we were unable to estimate divergence times due to the absence of calibrating points and we rely only on indirect evidence for our discussion. Irrespective of the exact time of the shift on Pinaceae, its occurrence further favoured the process of differentiation among *Thaumetopoea* moths. Indeed *Pinus* and *Cedrus* became successively isolated in the mountains of the Mediterranean region, during both interglacial and postglacial periods. The confinement to separated areas is a very likely factor speeding up the speciation, through geographic isolation, of *Thaumetopoea* taxa: range fragmentation due to biogeographical events and climatic changes during the late Tertiary, influencing host availability, has been invoked to explain allopatric speciation in clade B [38] and may also explain speciation in clade C.

The shift to winter feeding in clade B could be also interpreted as an escape from natural enemies, especially predators and parasitoids active during spring and summer. Although winter colonies are conspicuous and exposed for a long time to generalist predators such as insectivorous birds [87], the mortality is low because of the presence of urticating setae that protect the larvae from vertebrate predators [25]. The co/occurrence of social behaviour and defensive urticating setae in the *Thaumetopoea* larvae set them apart from the juvenile stages of all other Lepidoptera species. The advantages of sociality have been described for a number of organisms, including Lepidoptera [18]. In the case of the processionary moths, it has been shown that not only sociality *per se* but group size matters for colony performance and survival [78], [88], [89]. Foraging group may facilitate the attack of the host plant and at the same time lower the probability of being attacked by a predator. Moreover it could represent a further defensive strategy as aposematic signal for vertebrates [25]; noteworthy the only species showing a nomadic behaviour at the larval stage, *T. herculeana*, is also the less urticating species [20]. The evolutionary success was further enhanced by the pupation strategy that in most of the species occurs in the soil, while it is performed in the litter in *T. solitaria* or in a well-protected nest in *T. processionea*. This trait was involved for the different *Thaumetopoea* species in protection against vertebrate predation. Thus, the split in the three major clades can be viewed as the result of an adaptive radiation, made possible by an array of life history traits, which allowed a successful evolutionary response to changes in host use and climate.

## Supporting Information

Figure S1 **The secondary structure of** ***rrnL*** **domain I in Noctuoidea moths.**
(PDF)

Figure S2 **S2.1-S2.38. ML phylogenetic trees obtained from selected ALNs.**
(PDF)

Table S1 **PCR conditions used to amplify/sequence mitochondrial/nuclear genes.**
(PDF)

Table S2 **List of traits analyzed in the** ***Thaumetopoea*** **genus.**
(PDF)
