# Supplementary material for: Host and Phenology Shifts in the Evolution of the Social Moth Genus Thaumetopoea
Source: PLoS One. 2013 Feb 27;8(2):e57192. doi: 10.1371/journal.pone.0057192 (PMC3584119; doi:10.1371/journal.pone.0057192)
Supplement: Figure S1 — The secondary structure of rrnL domain I in Noctuoidea moths. (PDF) [file pone.0057192.s001.pdf]

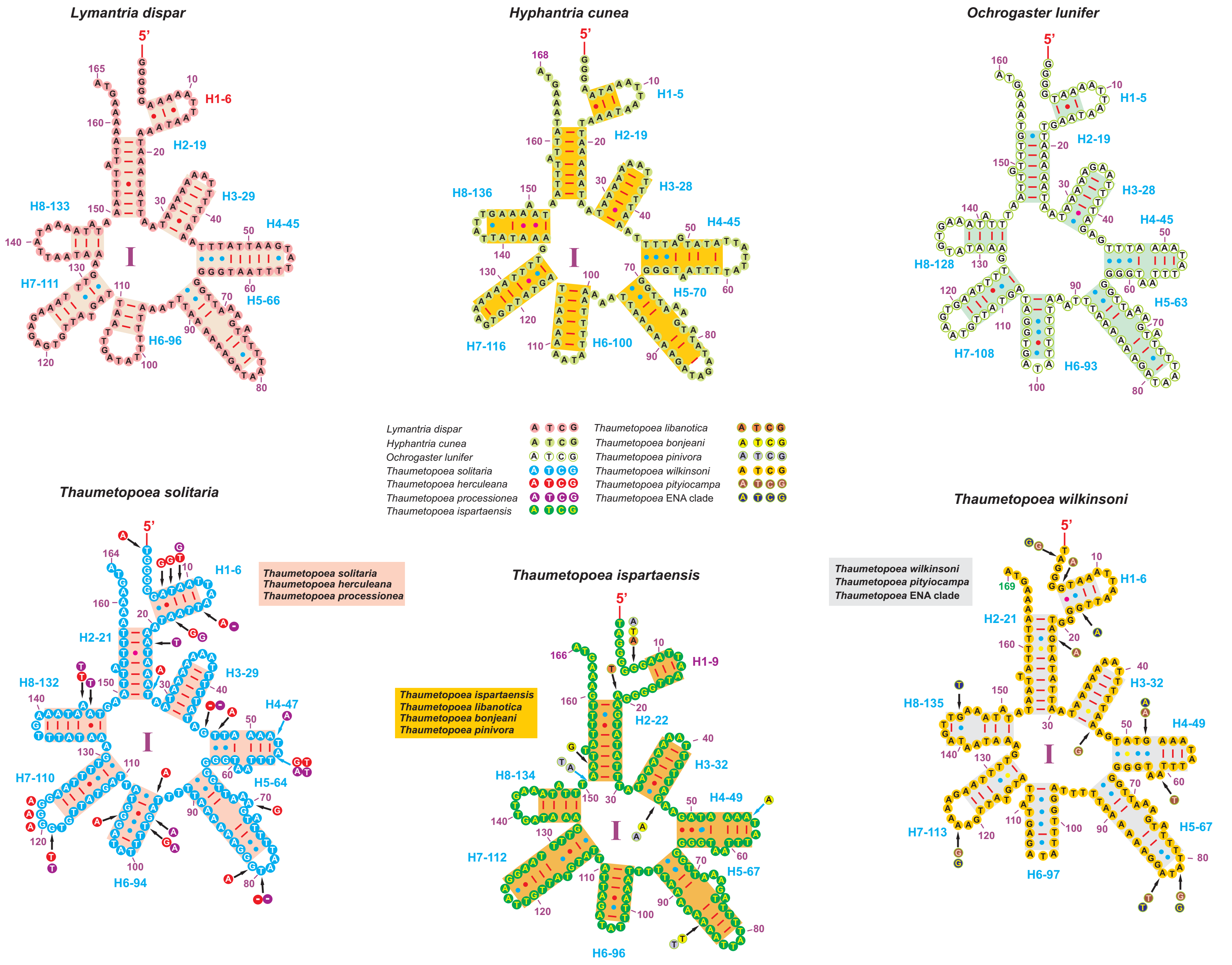

Figure S1. The secondary structure of *rrnL* domain I in Noctuoidea moths.
