# Supplementary material for: Host and Phenology Shifts in the Evolution of the Social Moth Genus Thaumetopoea
Source: PLoS One. 2013 Feb 27;8(2):e57192. doi: 10.1371/journal.pone.0057192 (PMC3584119; doi:10.1371/journal.pone.0057192)
Supplement: Figure S2 — S2.1-S2.38. ML phylogenetic trees obtained from selected ALNs. (PDF) [file pone.0057192.s002.pdf]

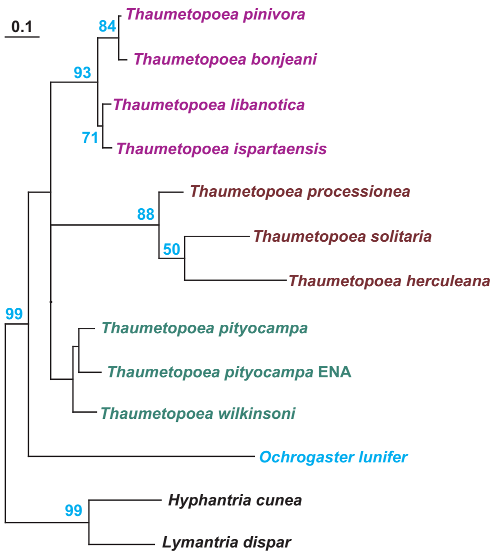

S.2.1. ML tree *atp8*

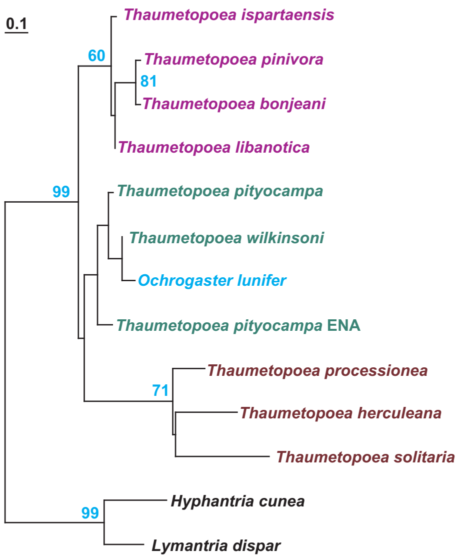

S.2.2. ML tree ATP8

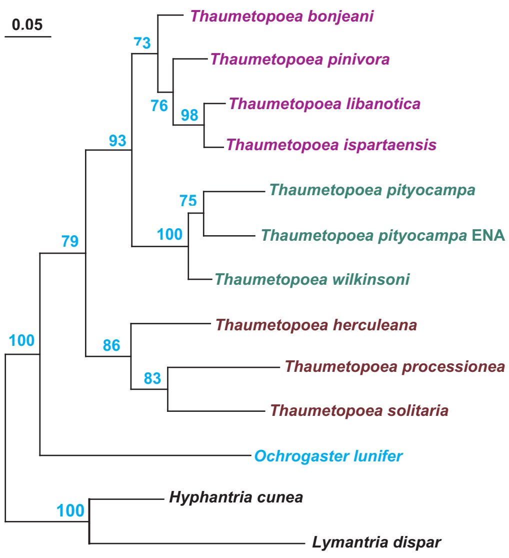

S2.3. ML tree *atp6*

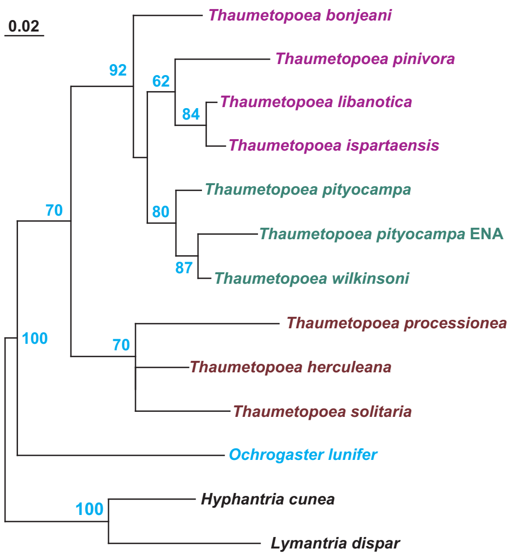

S2.4. ML tree ATP6

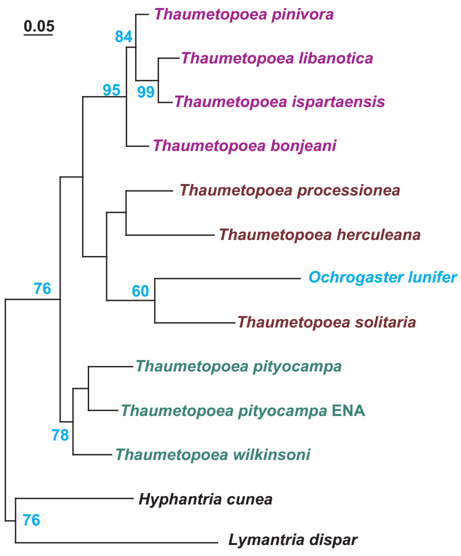

S2.5. ML tree *cob*

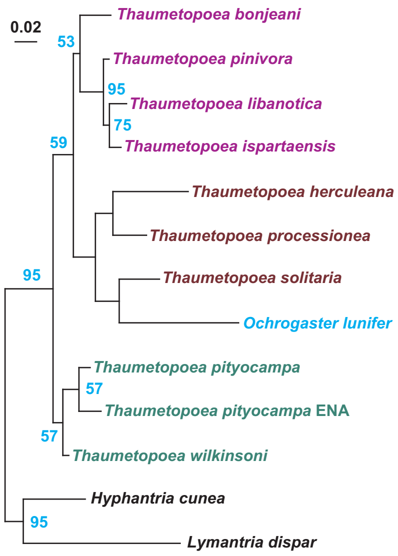

S2..6. ML tree CYTB

Figures S2.1-S2.38. ML phylogenetic trees obtained from selected ALNs.

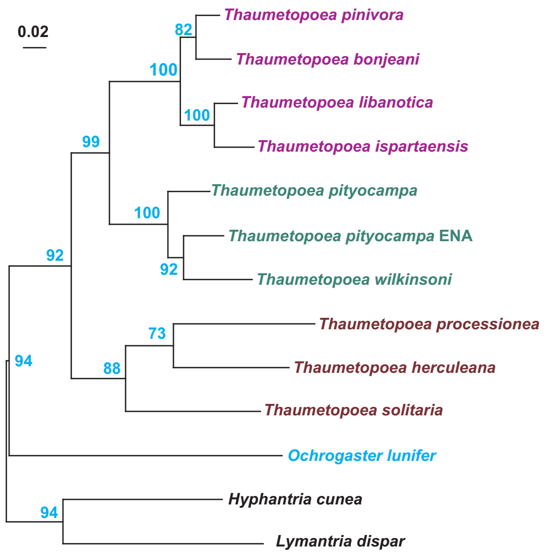

S2.7. ML tree *cox1*

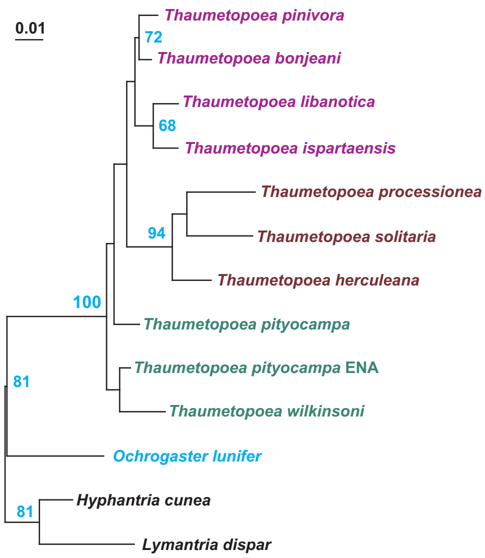

S2.8. ML tree COI

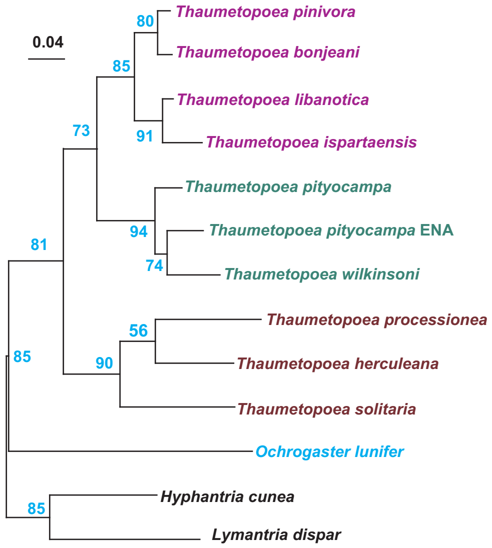

S2.9. ML tree *cox1*-BARCODE

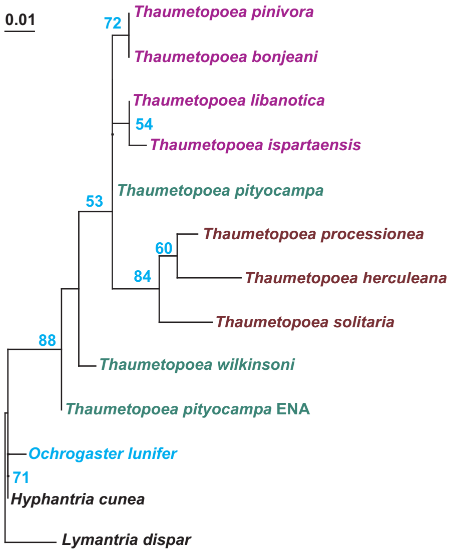

S2.10. ML tree COI-BARCODE

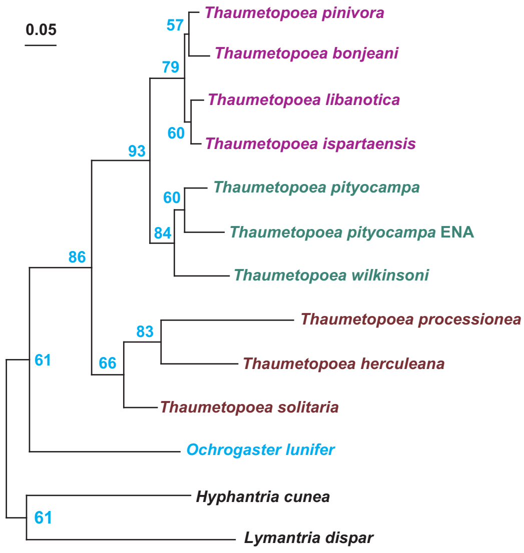

S2.11. ML tree *cox2*

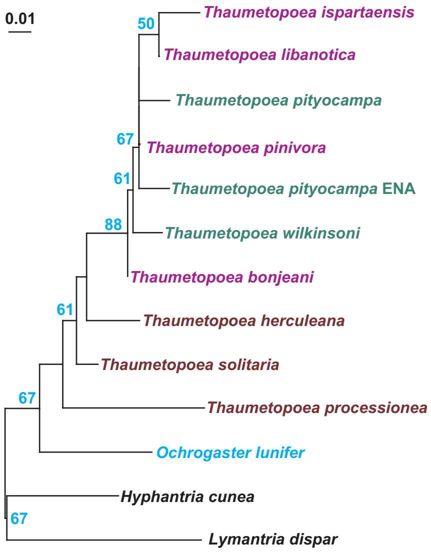

S2.12. ML tree COII

Figures S2.1-S2.38. ML phylogenetic trees obtained from selected ALNs.

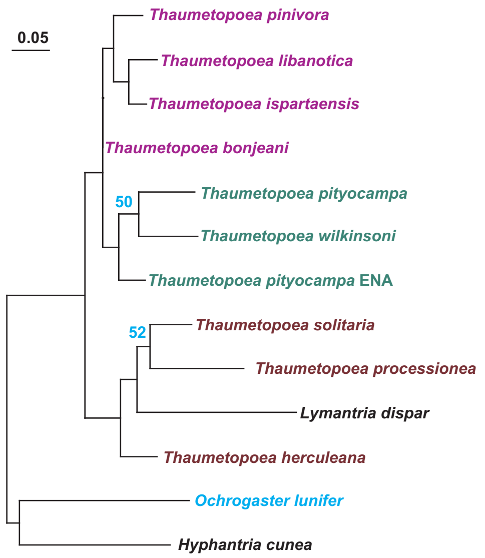

S2.13. ML tree *cox3*

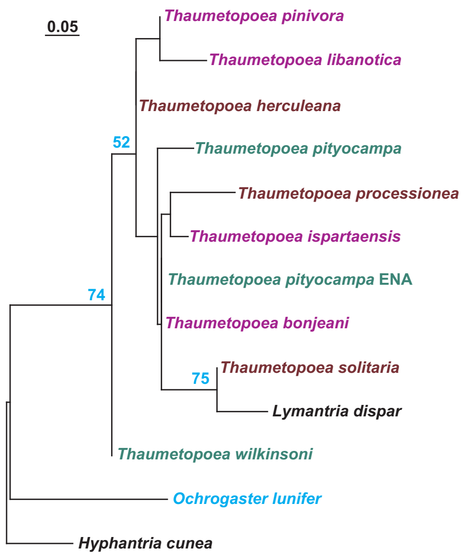

S2.14. ML tree COIII

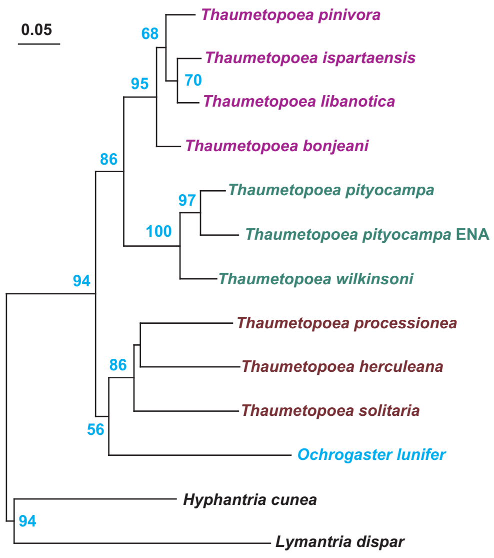

S2.15. ML tree *nad1*

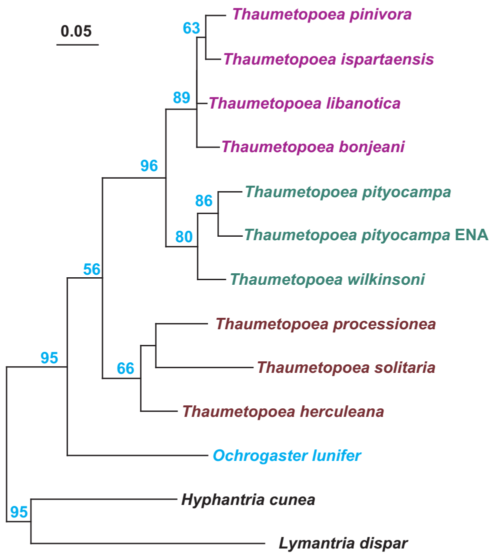

S2.16. ML tree NADH1

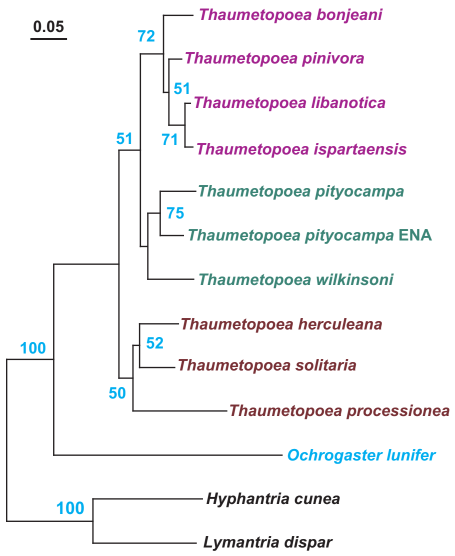

S2.17. ML tree *nad4*

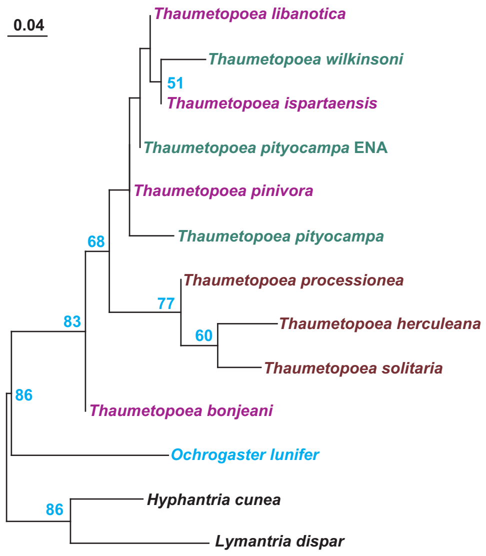

S2.18. ML tree NADH4

Figures S2.1-S2.38. ML phylogenetic trees obtained from selected ALNs.

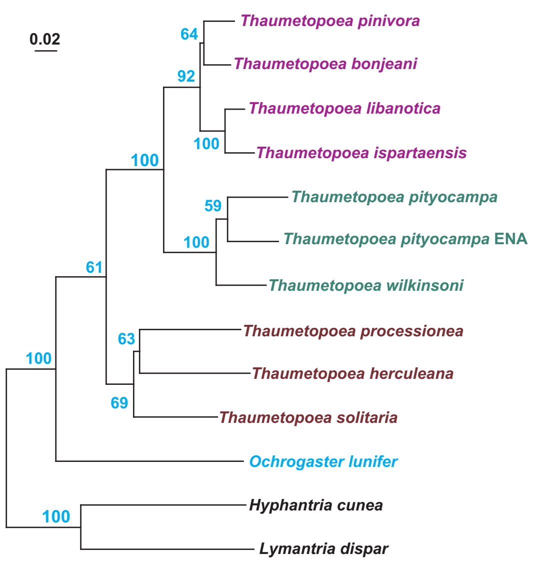

S2.19. ML tree *nad5*

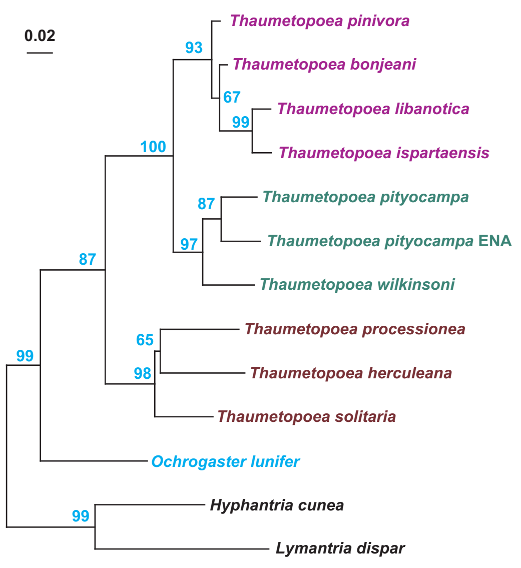

S2.20. ML tree NADH5

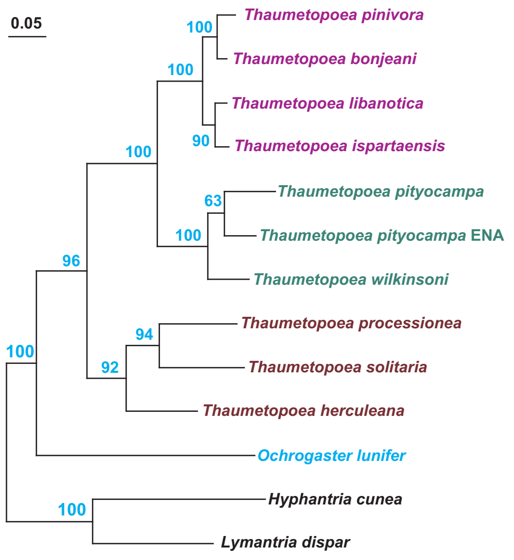

S2.21. ML tree *rrnL*

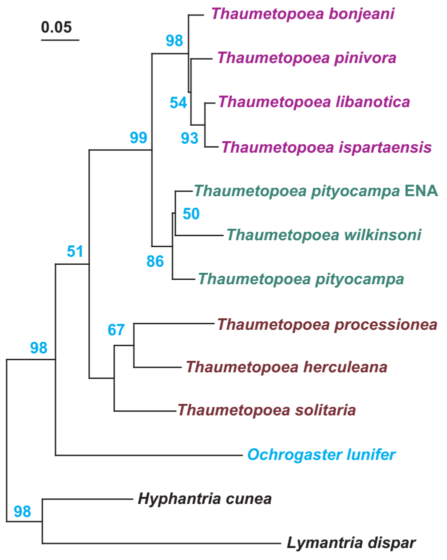

S2.22. ML tree *rrnS*

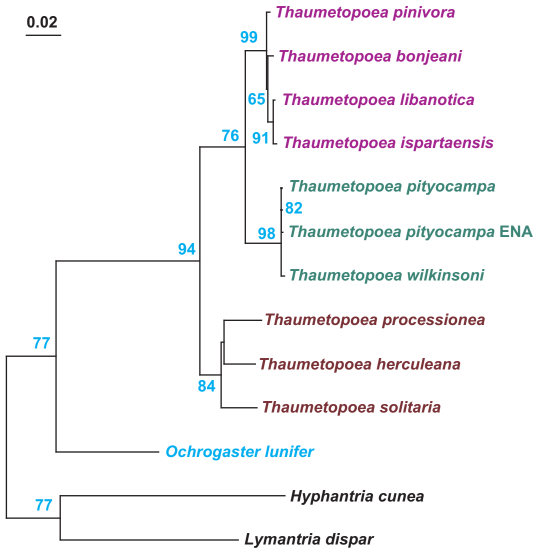

S2.23. ML tree *EF-1α*

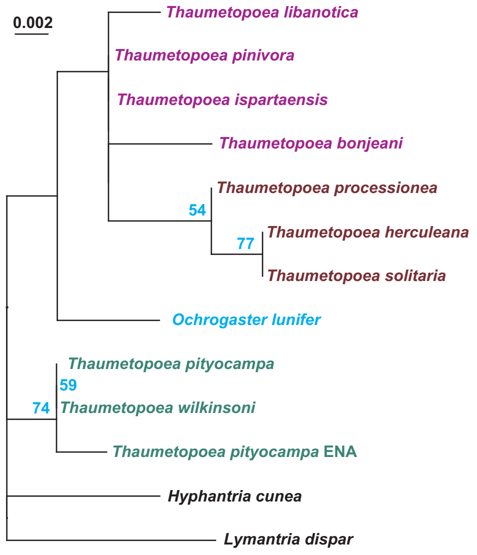

***S2.24.* ML tree EF-1A**

Figures S2.1-S2.38. ML phylogenetic trees obtained from selected ALNs.

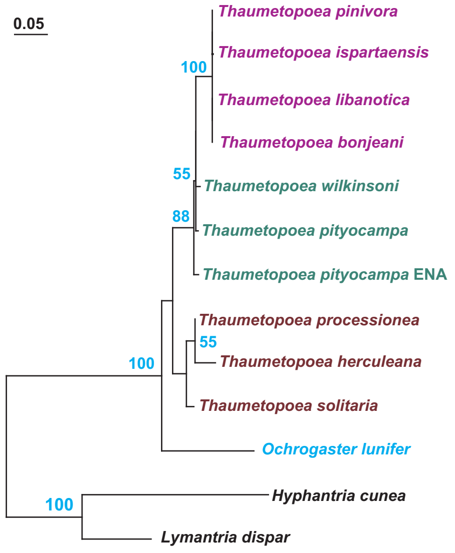

S2.25. ML tree *wng*

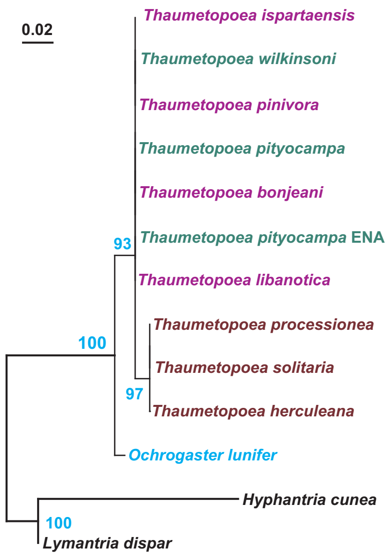

S2.26. ML tree WNG

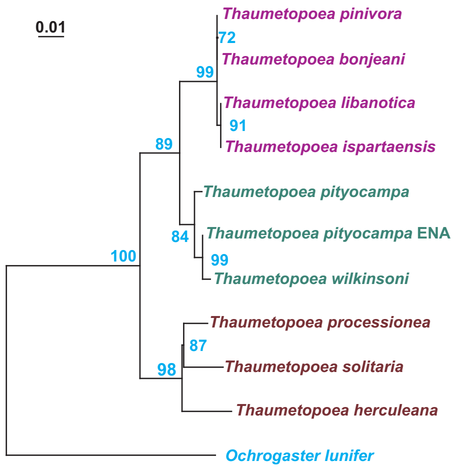

S2.27. ML tree *pho*

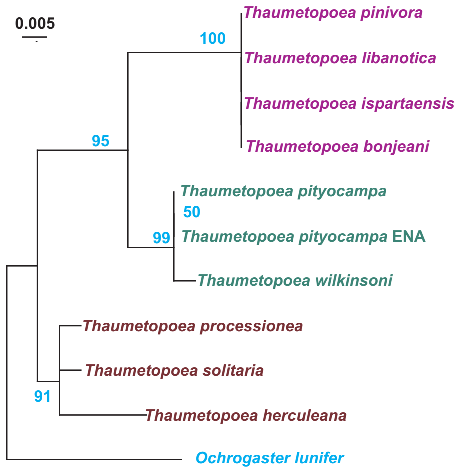

S2.28. ML tree PHO

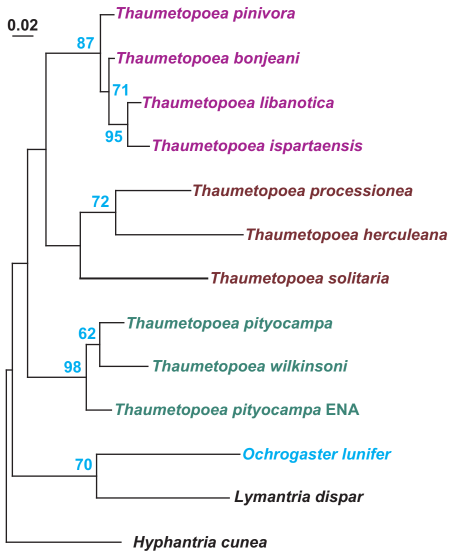

S2.29. ML tree *7trnas*

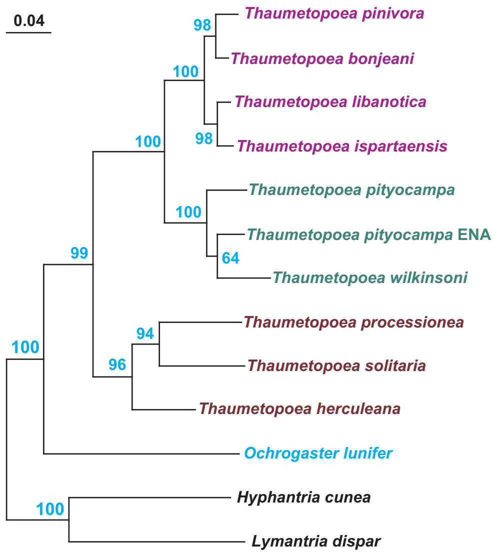

S2.30. ML tree *rrnL + rrnS*

Figures S2.1-S2.38. ML phylogenetic trees obtained from selected ALNs.

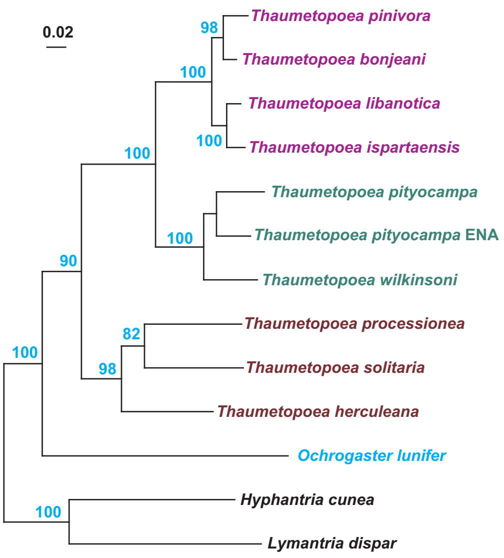

S2.31. ML tree *rrnL + rrnS + 7trnas*

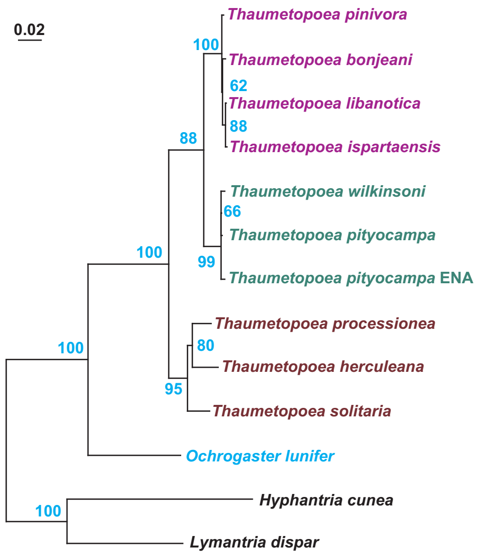

S2.32. ML tree *nuc2*

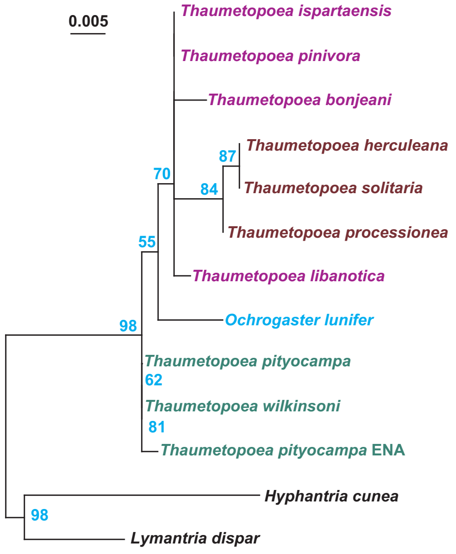

S2.33. ML tree NUC2

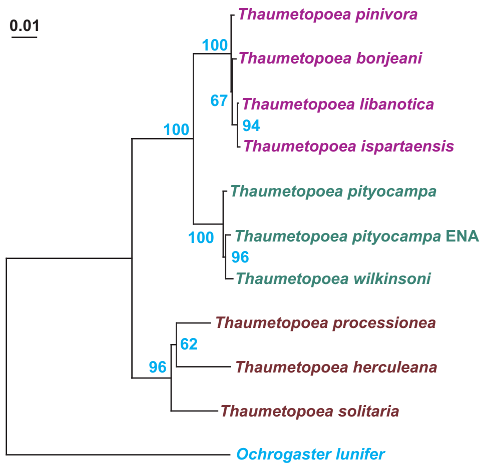

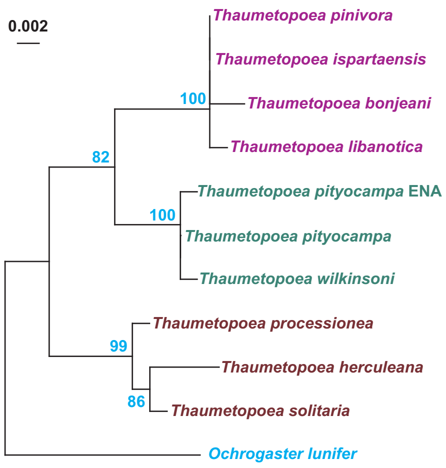

S2.34. ML tree *nuc3*

S2.35. ML tree NUC3

Figures S2.1-S2.38. ML phylogenetic trees obtained from selected ALNs.

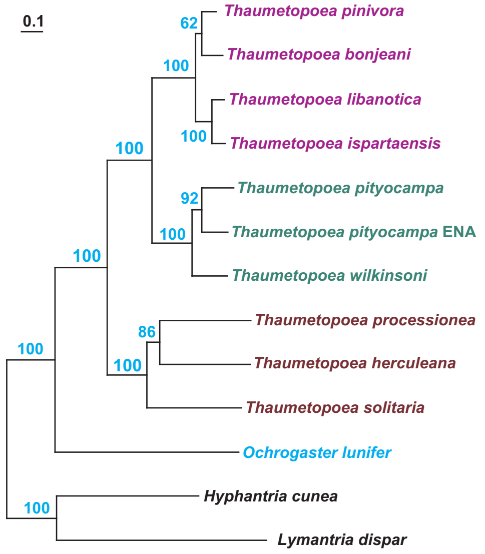

S2.36. ML tree *mtpcg*

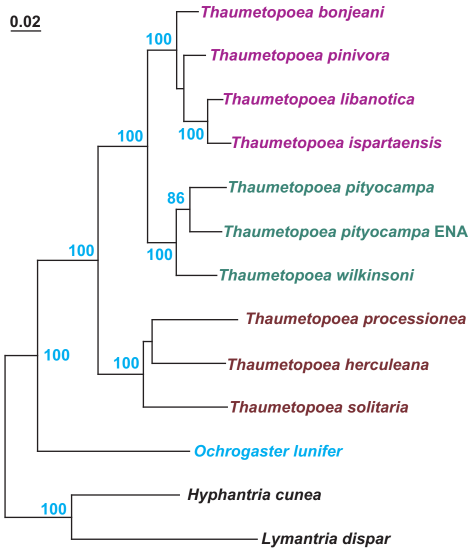

S2.37. ML tree mtPCG

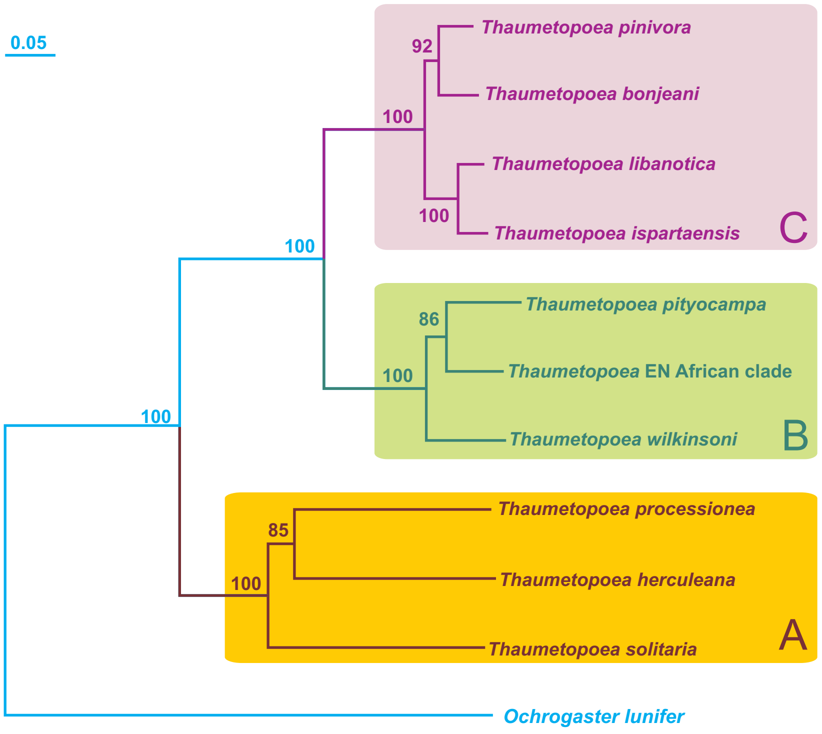

S2.38. ML tree aag*11th-set*

Figures S2.1-S2.38. ML phylogenetic trees obtained from selected ALNs.

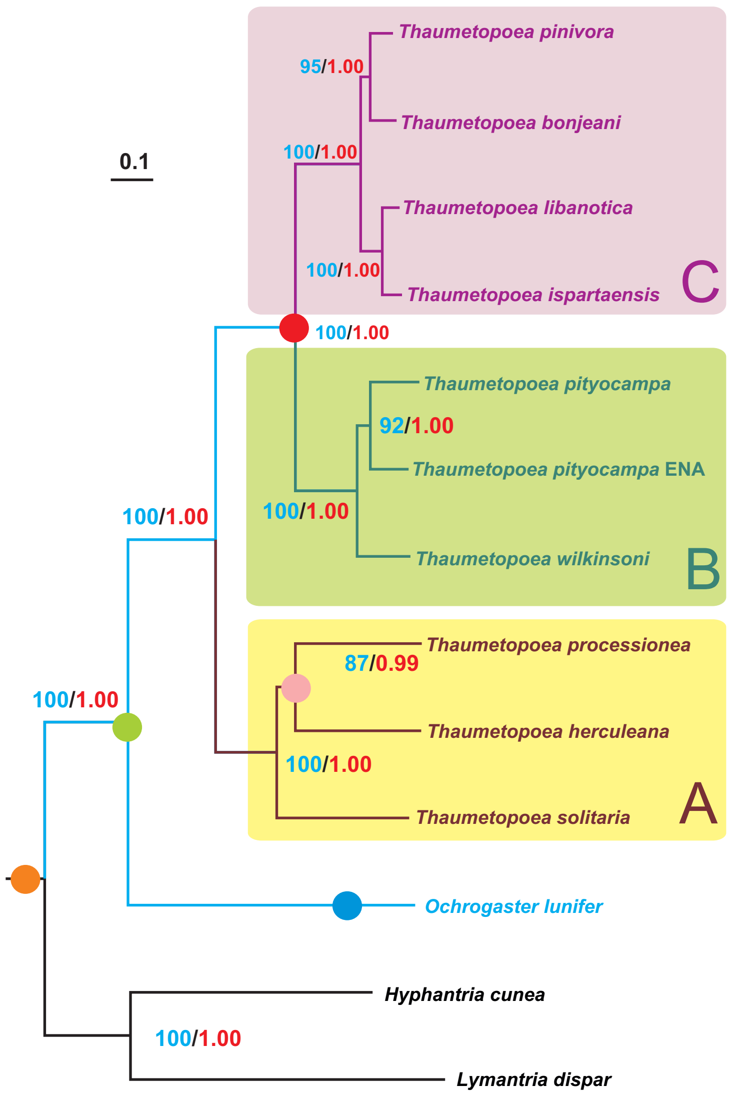

Figure 4. The reference phylogenetic tree.
Maximum likelihood tree (-ln = 44305.147421) inferred from the *aag13sp-set* ALN. The analysis was performed by applying the GTR+G evolutionary model and according to the most complex partitioning scheme described in the main text. Blue-coloured numbers indicate bootstap values expressed as percentage, whereas red-coloured numbers indicate posterior probabilities computed through Bayesian Inference analysis on the same data set. The scale bar represents 0.1 substitutions/site.

Brief comments to the trees obtained from various subsets described in the main text

Trees obtained from single gene/protein fully matched the reference topology shown above, or presented only a few discrepancies that were mostly restricted to the placement of single *Thaumetopoea* species within each major clade (Figs. S2.1-S2.28). Some ALNs (e.g. WNG, COIII) provided poorly resolved topologies, due to their limited phylogenetic signal. Bootstrap support for alternative placements was very variable ranging from good to marginal or absent (Figs. S2.1-S2.28). The same was true for ALNs concatenating several genes or proteins (Figs. S2.29-S2.38). In this case the discrepancies rather concern the placement of species within clades A-C. The alternative placements received variable statistical support (Figs. S2.29-S2.35). For example *rrnL+rrnS* favoured the sister group relationships: *T. solitaria* + *T. processionea* (BT = 94%) and *T. wilkinsoni* + *T. pityocampa* ENA (BT = 64%) (Fig. S2.30). In general, DNA ALNs produced better resolved topologies than amino acids ALNs.

Interestingly, the trees obtained either from the *cox1* gene and from the barcode region of this gene differed from the reference topology for the presence of the clade *T. pityocampa* ENA + *T. wilkinsoni* (rather than a clade *T. pityocampa* + *T. pityocampa* ENA) that received BT support (Figs. S2.7-S2.10). Moreover, the tree obtained from the barcode region only was poorly resolved.
