# Supplementary material for: Host and Phenology Shifts in the Evolution of the Social Moth Genus Thaumetopoea
Source: PLoS One. 2013 Feb 27;8(2):e57192. doi: 10.1371/journal.pone.0057192 (PMC3584119; doi:10.1371/journal.pone.0057192)
Supplement: Table S1 — PCR conditions used to amplify/sequence mitochondrial/nuclear genes. (PDF) [file pone.0057192.s003.pdf]

Table S1.A.1 PCR conditions used for mitochondrial genes

Thaumetopoea bonjeani

FW primer	RV primer	MIX	AT (°C)	EXT	n° cycles
TFJ6400	MPE_a_nad5	1	55	1'30"	35
allnd5form	MPE_a_nad5	1	50	1'45"	35
nad5pbihF	nad5pbihR	1	55	1'	35
N5J7572	N4N8727	1	53	1'45"	35
pf7925	pr8776	1	55	1'30"	35
CBJ10933	CBN11367	1	58	1'	35
CBJ10933	TS1N11683	1	52	1'	35
CBJ11485	N1N12442	1	54	1'30"	35
N1J12261m	LRN13000	2	52	1'	35
LRJ12887	LRN13398	1	58	1'	35
eochl10for	OC10rr	2	48	1'30"	35
SRJ14197	SRN14745	1	54	1'30"	35
SRJ14233	SRN14588	1	60	30"	35
pB12Sfor	pB12Srev	1	53	1'	35
LCO1490	HCO2198	1	50	1'	35
C1J1718	C1N2353	1	50	1'	35
C1J1751	TL2N3014	1	48	2'	35
allC1J1922	allC1N3120	1	52	1'30"	35
C1J2441	C2N3661	1	52	1'30"	35
C1J2792	TKN3796	1	49	1'30"	40
C2for2	A6N4552m	2	57	1'30"	35
C2J3624	C3N4908	1	52	1'30"	35

Thaumetopoea herculeana

FW primer	RV primer	MIX	AT (°C)	EXT	n° cycles
TFJ6400	MPE_a_nad5	1	55	1'30"	35
N5J7572	N4N8727	1	53	1'45"	35
nad5hwF	nad5hwR	1	55	1'	35
pf7925	pr8776	1	55	1'30"	35
CBJ10933	CBN11367	1	58	1'	35
ochlnad1for	allnd1rev	1	57	1'40"	35
CBJ11335	allnd1rev	1	57	1'40"	35
CBJ11545	LRN12866	1	57	1'30"	35
OC7rr	allnd1rev	1	54	1'30"	35
pth1612SAf	pth1612SAr	1	58	1'45"	35
N1J12261m	071105rev5	1	55	1'	35
pth1612SBf	pth1612SBr	1	58	1'45"	35
SRJ14197	TMN200	1	61	2'	35
SRJ14233	SRN14588	1	52	1'	30
LCO1490	HCO2198	1	50	1'	35
allC1J1922	allC1N3120	1	52	1'30"	35
C1J2441	TKN3785	1	50	2'	35

Table S1.A.2 PCR conditions used for mitochondrial genes

	FW primer	RV primer	MIX	AT (°C)	EXT	n° cycles
Thaumetopoea herculeana	**FW primer**	**RV primer**	**MIX**	**AT (°C)**	**EXT**	**n° cycles**
	C2J3399	A8N4061	1	50	1'	35
	TKJ3790	C3N4908	1	53	1'45"	35
	allco1for	allco3rev	1	61	2'30"	40
Thaumetopoea ispartaensis	**FW primer**	**RV primer**	**MIX**	**AT (°C)**	**EXT**	**n° cycles**
	TFJ6400	MPE_a_nad5	1	55	1'30"	35
	TFJ6400	N5N7793	1	54	1'45"	35
	allnd5form	MPE_a_nad5	1	50	1'45"	35
	N5J7572	N4N8727	1	53	1'45"	35
	tisp0710nd5f	tisp0710nd5r	1	52	45"	35
	nad5pbihF	nad5pbihR	1	55	1'	35
	pf7925	pr8776	1	55	1'30"	35
	CBJ10933	N1N12595	1	52	1'45"	40
	CBJ10933	allnd1rev	2	57	1'30"	35
	N1J12261m	LRN13000	2	52	1'	35
	LRJ12888	LRN13398	1	57	1'	35
	ochl16S12Sfor	OC10rr	1	62	1'15"	35
	LRJ12888	OC10rr	1	61	1'30"	35
	071105for1	071105rev2	1	55	1'30"	35
	LRJ13900	SRN14745	1	57	1'30"	35
	pB12Sfor	pB12Srev	1	53	1'	35
	LCO1490	HCO2198	1	50	1'	35
	C1J1718	C1N2353	1	50	1'	35
	C1J1718	C1N2776	1	52	1'	35
	allC1J1922	allC1N3120	1	52	1'30"	35
	C1J2441	TKN3785	1	50	2'	35
	C1J2441	C2N3661	1	52	1'30"	35
	C2J3399	A8N4061	1	48	1'	35
	C2J3624	C3N4908	1	52	2'	35
	eochl14for	eochl14rev	1	54	2'	35
	TKJ3790	C3N4908	1	62	2'	35
Thaumetopoea libanotica	**FW primer**	**RV primer**	**MIX**	**AT (°C)**	**EXT**	**n° cycles**
	TFJ6400	N5N7211m	1	55	1'45"	35
	allnd5form	MPE_a_nad5	1	50	1'45"	35
	pf7925	pr8776	1	55	1'30"	35
	N5J7572	N4N8727	1	53	1'45"	35
	N5J7806	N4N8727	1	57	1'45"	35
	CBJ10933	allnd1rev	2	57	1'30"	35
	CBJ10933	allcobrev	1	55	45"	35
	CBJ10933	N1N12595	1	52	1'45"	40

Table S1.A.3 PCR conditions used for mitochondrial genes

	FW primer	RV primer	MIX	AT (°C)	EXT	n° cycles
Thaumetopoea libanotica	CBJ11335	N1N12442	1	54	1'30"	35
	nd1micro	eochl7rev	1	58	45"	35
	N1J12261m	LRN13000	2	52	1'	35
	LRJ12888	LRN13398	1	57	1'	35
	LRJ12888	OC10rr	1	61	1'30"	35
	ochl16S12Sfor	OC10rr	1	62	1'15"	35
	SRJ14197	SRN14745	1	54	1'30"	35
	pB12Sfor	pB12Srev	1	53	1'	35
	071105for1	071105rev2	1	55	1'30"	35
	LCO1490	HCO2198	1	50	1'	35
	LCO1490	C1N2353	1	56	1'	35
	allC1J1922	allC1N3120	1	52	1'30"	35
	C1J2183	C2N3661	1	48	2'	35
	C1J2441	C2N3661	1	52	1'30"	35
	C1J2441	TKN3785	1	50	2'	35
	allco1for	allco3rev	1	61	2'30"	40
	TKJ3790	C3N4908	1	62	2'	35
Thaumetopoea pinivora	**FW primer**	**RV primer**	**MIX**	**AT (°C)**	**EXT**	**n° cycles**
	PRND51r	N4N8727	1	54	2'30"	35
	allnd5form	MPE_a_nad5	1	50	1'45"	35
	nad5pbihF	nad5pbihR	1	55	1'	35
	N5J7572	N4N8727	1	53	1'45"	35
	pf7925	pr8776	1	55	1'30"	35
	CBJ10933	CBN11367	1	58	1'	35
	nd1micro	12Srev2	1	50	1'	35
	OC7rr	allnd1rev	1	54	1'30"	35
	tpin0710cobf	tpin0710cobr	1	52	45"	35
	CBJ11335	N1N12442	1	54	1'30"	35
	CBJ11545	LRN12866	1	57	1'30"	35
	LRJ12887	LRN13398	1	58	1'	35
	LRJ12888	OC10rr	1	61	1'30"	35
	16Sfor2	12Srev2	1	50	1' 30"	35
	N1J12261m	LRN13000	2	52	1'	35
	ochl16S12Sfor	SRN14220	1	61	1'30"	35
	SRJ14233	SRN14588	1	52	1'	30
	LRJ13342	SRN14745	2	52	2'	35
	SRJ14197	TMN200	1	61	2'	35
	LCO1490	HCO2198	1	50	1'	35
	C1J1718	TL2N3014	1	48	1'30"	35
	allC1J1922	allC1N3120	1	52	1'30"	35

Table S1.A.4 PCR conditions used for mitochondrial genes

Thaumetopoea pinivora	FW primer	RV primer	MIX	AT (°C)	EXT	n° cycles
	C1J2183	TKN3772	1	52	2'	35
	C1J2441	TKN3785	1	50	2'	35
	C1J2441	C2N3661	1	52	1'30"	35
	allC2J3505	A6N4552m	1	55	1'30"	35
	A6J4463	COIIIrev	1	55	1'15"	35
	allco1for	allco3rev	1	61	2'30"	40
	C3if	C3ir	1	55	1'	35
	TpinA6C3for	TpinA6C3rev	1	50	1'	35
Theumetopoea pityocampa	**FW primer**	**RV primer**	**MIX**	**AT (°C)**	**EXT**	**n° cycles**
	TFJ6400	N5N7793	1	48	2'30"	35
	PRND51R	N4N8727	1	56	2'30"	30
	N5J7077	N4N8727	1	51	1'	35
	N5J7806	N4N8727	1	52	1'30"	35
	ochlNAD5for	N4N8727	1	48	45"	35
	CBJ10933	N1N12595	1	52	2'	35
	CBJ11335	N1N12442	1	52	1'	35
	CBJ11545	LRN12866	2	49	1'	35
	N1J12261m	LRN13000	1	62	2'	35
	ochlcobF1	LRN12866	1	56	1'45"	35
	ochlNAD1for	ochl16Srev	1	55	1'	35
	N1J12261m	SRN14220	2	50	1'45"	40
	N1J12585m	SRN14220	1	52	2'	35
	LRJ12888	SRN14220	1	55	1'30"	35
	LRJ13342	SRN14745	1	56	2'	35
	SRJ14197	TMN200	1	56	1'45"	35
	LCO1490	HCO2198	1	56	2'30"	30
	C1J1718	TL2N3014	1	53	2'	35
	C1J1718	C1N2353	1	56	1'45"	35
	C1J1751	C2N3661	1	53	2'	35
	allC1J1922	allC1N3120	1	53	2'	35
	C1J2183	C1N2659	1	61	1'30"	35
	C1J2441	C2N3661	1	54	1'30"	35
	C1J2441	TKN3785	1	57	2'	35
	C2J3399	A8N4061	1	55	1'	35
	allC2J3505	A8N4061	1	50	1'	30
	TKJ3790	Tpina6c3rev	1	54	2'30"	35
	C3if	C3ir	1	56	2'30"	30
	TKJ3790	C3N4908	1	58	45"	35

Table S1.A.5 PCR conditions used for mitochondrial genes

Thaumetopoea pityocampa ENA	FW primer	RV primer	MIX	AT (°C)	EXT	n° cycles
	ttun6600	ttun7806	1	54	1'15"	35
	allnd5form	MPE_a_nad5	1	50	1'45"	35
	allnd5form	allnd4rev	1	55	1'30"	35
	N5J7806	N4N8727	1	55	2'	35
	CBJ10612	CBN11010	1	58	1'30"	35
	tuncobforbis	tuncobrevbis	1	54	1'	35
	CBJ11335	allnd1rev	1	55	1'30"	35
	CBJ11545	N1N12595	1	53	1'30"	35
	CBJ11485	allnd1rev	1	55	1'30"	35
	ochl16S12Sfor	SRN14220	1	61	1'30"	35
	LRJ12887	LRN13398	1	58	1'	35
	ttun13206	ttun13740	2	60	1'	35
	LRJ13342	SRN14220	1	60	1'	35
	LRJ13900	SRN14588	1	60	1'	35
	LRJ13900	SRN14220	1	60	1'	35
	pther1612for	pther1612rev	1	54	1'30"	35
	SRJ14197	SRN14745	1	54	1'30"	35
	SRJ14197	TMN200	1	61	2'	35
	SRJ14610	TMN200	1	60	1'	35
	LCO1490	HCO2198	1	50	1'	35
	C1J1718	C1N2353	1	50	1'	35
	C1J1718	TL2N3014	1	55	1'30"	35
	allC1J1922	allC1N3120	1	52	1'30"	35
	C1J2792	TKN3796	1	49	1'30"	40
	TKJ3790	C3N4908	1	54	1'30"	35
	ttun3900	ttun4540	2	60	1'	35

Thaumetopoea processionea	FW primer	RV primer	MIX	AT (°C)	EXT	n° cycles
	TFJ6400	trnand5rev	1	53	2'	35
	PRND51R	N4N8727	1	54	2'30"	35
	N5J7077	N4N8727	1	51	1'45"	35
	allnd5form	MPE_a_nad5	1	50	1'45"	35
	N5J7572	N4N8727	1	53	1'45"	35
	pf7925	pr8776	1	55	1'30"	35
	CBJ10612	CBN11010	1	58	1' 30"	35
	CBJ10933	TS1N11683	1	52	1'	30
	tpea_0710nd5f	tpea_0710nd5r	1	55	1'	35
	CBJ11335	N1N12442	1	55	1' 30"	35
	tpea_0710cob2f	tpea_0710cob2r	1	55	1'	35
	N1J12261m	LRN12866	1	61	2'	35
	N1J12261m	LRN13000	2	52	2'	35

Table S1.A.6 PCR conditions used for mitochondrial genes

Thaumetopoea processionea	FW primer	RV primer	MIX	AT (°C)	EXT	n° cycles
	LRJ12887	LRN13398	1	52	1'	30
	LRJ12888	12Srev2	1	60	1'45"	33
	16Sfor2	12Srev2	1	50	1' 30"	35
	SRJ14197	TMN200	1	61	2'	35
	SRJ14233	SRN14588	1	52	1'	30
	LCO1490	HCO2198	1	52	1'	30
	allC1J1922	allC1N3120	1	52	1'30"	35
	C1J2183	TKN3772	1	52	2'	35
	C1J2441	TKN3785	1	48	2'	35
	C2J3399	A8N4061	1	52	1'	35
	TKJ3790	C3N4908	1	62	2'	35
Thaumetopoea solitaria	**FW primer**	**RV primer**	**MIX**	**AT (°C)**	**EXT**	**n° cycles**
	TFJ6400	MPE_a_nad5	1	55	1'30"	35
	allnd5form	MPE_a_nad5	1	50	1'45"	35
	pf7925	pr8776	1	55	1'30"	35
	N5J7572	N4N8727	1	53	1'45"	35
	N53f	N4N8727	1	50	1'30"	35
	CBJ11485	N1N12442	1	50	1'30"	35
	OC7rr	allnd1rev	1	54	1'30"	35
	nd1micro	eochl7rev	1	55	45"	35
	N1J12261m	LRN13000	2	52	1'	35
	LRJ12888	LRN13398	1	57	1'	35
	LRJ12888	OC10rr	1	61	1'30"	35
	eochl9for	OC10rr	1	54	1'15"	35
	tsol16Sfor	tsol16Srev	1	58	1'	35
	LCO1490	HCO2198	1	50	1'	35
	SRJ14197	TMN200	2	61	1'30"	35
	C1J1751	C1N2659	1	52	1'30"	35
	allC1J1922	allC1N3120	1	52	1'30"	35
	C1J2441	TKN3785	1	50	2'	35
	C1J2441	C2N3661	1	52	1'30"	35
	allC2J3505	allC3N4923	2	55	1'30"	35
	C3if	C3ir	1	55	1'	35
	TKJ3790	C3N4908	1	62	2'	35
Thaumetopoea wilkinsoni	**FW primer**	**RV primer**	**MIX**	**AT (°C)**	**EXT**	**n° cycles**
	PRND51R	N4N8727	1	54	2'30"	35
	allnd5form	MPE_a_nad5	1	50	1'45"	35
	nad5hwF	nad5hwR	1	55	1'	35

Table S1.A.7 PCR conditions used for mitochondrial genes

Thaumetopoea wilkinsoni	FW primer	RV primer	MIX	AT (°C)	EXT	n° cycles
	N53f	N4N8727	1	50	1'30"	35
	N5J7572	N4N8727	1	53	1'45"	35
	twilki0710nd5f	twilki0710nd5r	1	52	1'	35
	CBJ10933	allnd1rev	2	57	1'30"	35
	CBJ11335	allnd1rev	1	55	1'30"	35
	OC7rr	allnd1rev	1	54	1'30"	35
	CBJ11545	LRN12866	1	57	1'30"	35
	N1J12261m	LRN13000	2	52	1'	35
	twilki0710cobf	twilki0710cobr	1	52	1'	35
	LRJ12888	LRN13398	1	57	1'	35
	ochl16S12Sfor	OC10rr	1	62	1'15"	35
	SRJ14197	TMN200	1	61	2'	35
	LCO1490	HCO2198	1	50	1'	35
	C1J1718	TL2N3014	1	48	1'30"	35
	allC1J1922	allC1N3120	1	52	1'30"	35
	C1J2441	TKN3785	1	50	2'	35
	C1J2441	C2N3661	1	52	1'30"	35
	twilki0710cox1f	twilki0710cox1r	1	52	45"	35
	C1J2441	TKN3785	1	50	2'	35
	eochl14for	eochl14rev	1	54	2'30	35
	C2J3399	A8N4061	1	48	1'	35
	TKJ3790	C3N4908	1	62	2'	35

Table S1.A.8 PCR conditions used for mitochondrial genes

FW primer	5'-sequence-3'	RV primer	5'-sequence-3'
071105for1	ACTTTTGTATAACCGCAACT	071105rev2	AATAAGCACAATAGTTTTTGA
16Sfor2	GCAGCCCTTCAATCAA	071105rev5	TAGTACGAAAGGAATGAATA
A6J4463	TTTATTCATATWATWCCNCAAGG	12Srev2	TATTGCCCGTCGCTTT
allC1J1922	GGAATTTCHTCHATTTTAGG	A6N4552m	ATGTCCTGCAATTATATTWGC
allC2J3505	TTTTACCMATAAACAAYCAAATTC	A8N4061	GAAAATAAGTTWGTTATCATTTTCA
allco1for	CAGCTGAACATTCYTATAATGA	allC1N3120	AAAATAATTTGTTCTATTAARGG
allnd5form	ATTCTCTTCAACCBAAATCAATAY	allC3N4923	TTTACCTTGAAGDGTTCCTTCTCG
C1J1718	GGAGGATTTGGAAATTGATTAGTTCC	allco3rev	AWAYATCTCGYCATCATTGRTA
C1J1751	GGATCACCTGATATAGCATTCCC	allcobrev	GGRATAGATCGRAGAATTGC
C1J2183	CAACATTTATTTTGATTTTTTGG	allnd1rev	RCARCCTTTTTCWGATGCTATT
C1J2441	CCTACAGGAATTAAAATTTTTAGATGATTAGC	allnd4rev	AATTATTGGGGSTATRTKGGTTC
C1J2792	ATACCTCGACGTTATTCAGA	C1N2353	GCTCGTGTATCAATATCTATWCC
C2for2	CCCATCAATAGGAGTAAAAG	C1N2659	GCTAATCCAGTGAATAATGG
C2J3399	TCTATCGGACAYCAATGATAYTG	C1N2776	GATAATCTGAATATCGWCGNGG
C2J3624	AATCCAGGTCGATTAAAYCA	C2N3661	CCACAAATTTCTGAACATTGACCA
C3if	TATCAAAAGGATTACGATGAGG	C3ir	CGTCAACAAAGTGTCAATATCA
CBJ10612	CCATCCAACATCTCAGCATGATGAAA	C3N4908	CGTGAAAYATCTCGTCATCATTG
CBJ10933	TATGTACTACCATGAGGACAAATATC	CBN11010	TATCTACAGCAAATCCCCCTCA
CBJ11335	CAYATTCAACCTGAATGATA	CBN11367	ATTACACCTCCTAATTTATTAGGAAT
CBJ11485	TTCAATTTTACCCTATTAAYCA	CBN11526	TTCTACAGGTCGRGCTCCGATYCA
CBJ11545	ACATGAATTGGAGCTCGACCAGT	COIIIrev	GACCATGAAATCCTGTTGCTAT
eochl10for	TCCAATCTTTCATACAAGTCA	eochl14rev	CAGCCTGATATTGGCATT
eochl14for	TTCCCCTATATTTTCACCAG	eochl7rev	AAAAAGACGAGAAGACCCTA
eochl9for	TAAATATTTGCCGAATTCCT	HCO2198	TAAACTTCAGGGTGACCAAAAAATCA
LCO1490	GGTCAACAAATCATAAAGATATTGG	LRN12866	ACATGATCTGAGTTCAAACCGG
LRJ12887	CCGGTCTGAACTCAGATCACGT	LRN13000	TTACCTTAGGGATAACAGCGTAA
LRJ12888	CCGGTTTGAACTCARATCATGTA	LRN13398	CCCTTGTTTATCAAAAACAT
LRJ13342	CCTTTGTACRGTCAAAATACYGC	MPE_a_nad5	CCTGTTTCTGCTTTAGTTCA
LRJ13900	TTTAATAAACYCTGATACAMAAG	N1N12442	AATTTGRGKATTAATTCCTT
N1J12261m	TACTTCGTAAGAAATTGTTTGAGC	N1N12595	GTAGCATTTTTAACTTTATTAGAACG
N1J12585m	GGTCCTTTACGAATCTGAATATAACCT	N4N8727	AAATCTTTRATTGCTTATTCWTC
N53f	GCAGAAATACCAGCCATA	N5N7211m	TTAAAGCATTATTGTTTATGTGTGC
N5J7077	TTAAATCTTTWGARTAAAAYCC	N5N7793	TTGGGTTGRGATGGNYTAGG
N5J7572	AAAGGAATTTGAGCTCTTTTWGT	nad5hwR	TGGTTTGGGRTTRGTTTCTT
N5J7806	GAAACTAAACCCAAACCATCTCA	nad5pbihR	CATGGTTTATGTTCTTCTGG
nad5hwF	TAAAAMCCAGCTAAAAAWGG	OC10rr	TTAAATCAAATCAAGATGCAGA
nad5pbihF	AAATACACYCCCCCAATAAA	ochl16Srev	TAGATACGTGGTTTGTTGATCC
nd1micro	CCAACTAAAACCCCTAAAA	pB12Srev	AATTTTGGTGTTAAATAATTC
OC7rr	ATGCAACATTAACACGATTT	pr8776	TTTGTGGKGTCAAAAATATG
ochl16S12Sfor	TTTCATCCAATCTTTCATACAA	pth1612SAr	AGTCTAATCTGCCCMCTGAT
ochlcobF1	TTCCACCCATATTTTTCCTTTA	pth1612SBr	AAATCAAATCAAGATGCAGA
ochlNAD1for	TAACTCTCTCTCACCTTCAGCA	pther1612rev	TTTTATATTTTAGGGGATAAGC

Table S1.A.10 PCR conditions used for mitochondrial genes

FW primer	5'-sequence-3'	RV primer	5'-sequence-3'
ochlNAD5for	AGCAGAAATACCAGCCATAAGT	SRN14220	ATATGYACAYATTGCCCGTC
pB12Sfor	AATAGGGTATCTAATCCTAGTTT	SRN14588	AAACTAGGATTAGATACCCTATTAT
pf7925	TTGTCTCAAAGTAGATAAAGCAA	SRN14745	GTGCCAGCAGYYGCGGTTANAC
PRND51r	ATGGAATTCCACATAAAGCTAT	tisp0710nd5r	ATGGTTGGTCATGGCTTATG
pth1612SAf	AAAATAAATCTCACCACAGG	TKN3772	GAGACCATTACTTGCTTTCAGTCATCT
pth1612SBf	AATCTTTCATWCAAGTCAYCAA	TKN3785	GTTTAAGAGACCAGTACTTG
pther1612for	AATCCTTCATTCAAGTCACC	TKN3796	ACTATAAAATGGTTTAAGAG
SRJ14197	GTACCYCTACTTTGTTACGACTT	TL2N3014	TCCAATGCACTAATCTGCCATATTA
SRJ14233	AAGAGCGACGGGCGATGTGT	TMN200	TCCTTTATATTTGAGGTATGARCC
SRJ14610	ATAATAGGGTATCTAATCCTAGT	tpea_0710nd5r	GCTGCAATAACTAAAAGAGC
TFJ6400	TAATATCTTCAATRTYAARCTCT	tpea0710cob2r	AGTTTGAAATGAAATGTTAATCG
tisp0710nd5f	CTCATATTTTTGACCCCACA	tpin0710cobr	AAAGTCTAATCTGCCCACTGA
TKJ3790	CATTAGATGACTGAAAGCAAGTA	tpina6c3rev	TCTTAAAATGGCAATAACATAAGA
tpea0710cob2f	ATCAGGGGGCAGATTAGACT	trnand5rev	AACTCATGCGATATTTAAAGC
tpea0710nd5f	GAAAATAGGAACCACCCTAA	TS1N11683	TATTTCTTTATTATGTTTTCAAAAC
tpin0710cobf	CCAATTAAAACCCCCAAAA	tsol16Srev	TTTGAAATGAAATGTTAATCGT
tpina6c3for	TCCCTTTCAATTTCTTTACCA	ttun13740	TTAAAAAGACGAGAAGACCCTA
tsol16Sfor	TTACGCTGTTATCCCTAAGGT	ttun4540	CATAATGGCAGAGAAATTGAGA
ttun13206	ATTAGAGGATCAACCAGCAACT	ttun7806	TTTTTGGAGTGGGAAATTATTA
ttun3900	TTGCATTACCATCATTACGACT	tuncobrevbis	TTGTCCTGTAATAATGTAAGGTTC
ttun6600	TGATAACCCCAGCACATATAAA	twilki0710cobr	ACAACCTTTTTCAGATGCTA
tuncobforbis	AGGAACAGCCTTCATAGGAT	twilki0710cox1r	AGCTCCTAAAATGGAGGAAA
twilki0710cobf	AGCCCGACCTGTTGAAGAC	twilki0710nd5r	AGTAAGATTTGTGATGGGAAT
twilki0710cox1f	TTGTAACAGCCCATGCTTT		
twilki0710nd5f	AGTAGGAGCAGCCATAGCC		

	Mix 1	Mix 2
**Buffer 10X**	**4µl**	**4µl**
**MgCl2 25mM**	**2µl**	**1,5µl**
**dNTP's 2mM**	**1µl**	**1µl**
**primer fw 10µM**	**1µl**	**0,8µl**
**primer rw 10µM**	**1µl**	**0,8µl**
**Taq (5U/µl)**	**0,2µl**	**0,2µl**
**DNA sample**	**2µl**	**2µl**
**H2O**	**8,8µl**	**9,7µl**
**TOTAL**	**20µl**	**20µl**

AT = annealing Temperature in °C

EXT = Extension Time

Table S1.B.1 PCR conditions used for nuclear genes

	gene	forward primer	reverse primer	AT (°C)	EXT	n° cycles
Ochrogaster lunifer	**gene**	**forward primer**	**reverse primer**	**AT (°C)**	**EXT**	**n° cycles**
	EF	cho	EFrcM4	61	1'15"	30
		EF1	EF4b	51	2'	35
	wingless	LepWG1	LepWG2	61	1'15"	30
Thaumetopoea bonjeani	**gene**	**forward primer**	**reverse primer**	**AT (°C)**	**EXT**	**n° cycles**
	EF	cho	EFrcM4	61	1'15"	30
		starsky	luke	50	1'	35
		tbonEFfor	tbonEFrev	60	1'	35
	wingless	LepWG1	LepWG2	62	1'15"	35
Thaumetopoea herculeana	**gene**	**forward primer**	**reverse primer**	**AT (°C)**	**EXT**	**n° cycles**
	EF	Cho	EFrcM4	61	1'15"	35
		EF3	EFrcM4	53	1'30"	35
		Efbtshfor	EFrcM4	63	1'	35
		pEF1for	pEF1rev	54	50"	35
		PtbonEFfor	EF4b	55	1'	35
	wingless	LepWG1	LepWG2	62	1'15"	35
Thaumetopoea ispartaensis	**gene**	**forward primer**	**reverse primer**	**AT (°C)**	**EXT**	**n° cycles**
	EF	cho	EFrcM4	61	1'15"	30
		starsky	luke	50	1'	35
	wingless	LepWG1	LepWG2	61	1'15"	30
Thaumetopoea libanotica	**gene**	**forward primer**	**reverse primer**	**AT (°C)**	**EXT**	**n° cycles**
	EF	cho	EFrcM4	61	1'15"	30
		Plibpit5EFfor	Plibpit5EFrev	55	1'	35
	wingless	LepWG1	LepWG2	61	1'15"	30
Thaumetopoea pinivora	**gene**	**forward primer**	**reverse primer**	**AT (°C)**	**EXT**	**n° cycles**
	EF	cho	EFrcM4	61	1'15"	30
		starsky	luke	50	1'	35
	wingless	LepWG1	LepWG2	61	1'15"	30
Thaumetopoea pityocampa	**gene**	**forward primer**	**reverse primer**	**AT (°C)**	**EXT**	**n° cycles**
	EF	cho	EFrcM4	61	1'15"	30
		Plibpit5EFfor	Plibpit5EFrev	55	1'	35
	wingless	LepWG1	LepWG2	61	1'15"	30
***Thaumetopoea pityocampa* ENA**	**gene**	**forward primer**	**reverse primer**	**AT (°C)**	**EXT**	**n° cycles**
	EF	Cho	EFrcM4	61	1'15"	35
		Plibpit5EFfor	Plibpit5EFrev	63	45"	35
	wingless	LepWG1	LepWG2	61	1'15"	30

Table S1.B.2 PCR conditions used for nuclear genes

	gene	forward primer	reverse primer	AT (°C)	EXT	n° cycles
Thaumetopoea processionea						
	EF	cho	EFrcM4	61	1'15"	30
		EF1	EF4b	51	2'	35
		Efbtshfor	EFrcM4	63	1'	35
	wingless	LepWG1	LepWG2	61	1'15"	30
Thaumetopoea solitaria	**gene**	**forward primer**	**reverse primer**	**AT (°C)**	**EXT**	**n° cycles**
	EF	EF1	pEF1rev	53	1'30"	35
		Plibpit5EFfor	pEF1rev	58	1'	35
		PtbonEFfor	EF4b	55	1'	35
	wingless	LepWG1	LepWG2	61	1'15"	30
Thaumetopoea wilkinsoni	**gene**	**forward primer**	**reverse primer**	**AT (°C)**	**EXT**	**n° cycles**
	EF	cho	EFrcM4	61	1'30"	30
		Plibpit5EFfor	Plibpit5EFrev	55	1'	35
	wingless	LepWG1	LepWG2	61	1'15"	30

Table S1.B.3 PCR conditions used for nuclear genes

**Primer forward**	
**Cho**	GTCACCATCATYGACGC
**EF1**	GGCCACTTGATCTACAAAT
**EF3**	TCAAGAAAATYGGTTATAAC
**Efbtshfor**	GGAYTCCACTGAGCCCCCWT
**LepWG1**	GARTGYAARTGYCAYGGYATGTCTGG
**pEF1for**	ACYGGYCACTTGATCTAC
**Plibpit5EFfor**	CGACTCCGGCAAGTCCAC
**PtbonEFfor**	TTGAGGAAATCAAGAAAGAAG
**starsky**	CACATYAACATTGTCGTSATYGG
**tbonEFfor**	CCCTGCTTGCTTTTACACTT
**Primer reverse**	
EFrcM4	ACAGCVACKGTYTGYCTCATRTC
luke	CATRTTGTCKCCGTGCCAKCC
EF4b	GAGAGGAGGGAATTCTTG
pEF1rev	AACAATAGTACCAGGCTTGAG
Plibpit5EFrev	AGCTGCTTTACACCAAGTG
tbonEFrev	TGCATCTCCACAGACTTGAC
LepWG2	ACTCGCARCACCARTGGAATGTRCA

**Mix**	**AT = annealing Temperature in °C**
	**EXT = Extension Time**
**Buffer 10X**	**4µl**
**MgCl2 25mM**	**2µl**
**dNTP's 2mM**	**1µl**
**primer fw 10µM**	**1µl**
**primer rw 10µM**	**1µl**
**Taq (5U/µl)**	**0,2µl**
**DNA sample**	**2µl**
**H2O**	**8,8µl**
**TOTAL**	**20µl**
