# Supplementary material for: Host and Phenology Shifts in the Evolution of the Social Moth Genus Thaumetopoea
Source: PLoS One. 2013 Feb 27;8(2):e57192. doi: 10.1371/journal.pone.0057192 (PMC3584119; doi:10.1371/journal.pone.0057192)
Supplement: Table S2 — List of traits analyzed in the Thaumetopoea genus. (PDF) [file pone.0057192.s004.pdf]

Table S2.A. List of traits analysed in *Thaumetopoea* genus. Species used for the analysis (1st part).

Trait	*T. bonjeani*	*T. herculeana*	*T. ispartaensis*	*T. libanotica*	*T. pinivora*	*T. pityocampa*	*T. pityocampa* ENA
**Biogeography**							
distribution	localized	localized	localized	localized	localized	widespread	widespread
region	North West Africa	West Mediterranean	East Mediterranean	East Mediterranean	North Europe, West Europe	South Europe, North-West Africa, West Asia	North-Eeast Africa
zoogeographic distribution	Mediterranean	Mediterranean	Mediterranean, Iranoturanic	Mediterranean, Iranoturanic	Eurosibiric	Mediterranean, Eurosibiric	Mediterranean
**Host plant**							
host group	Gymnosperm	Angiosperm	Gymnosperm	Gymnosperm	Gymnosperm	Gymnosperm	Gymnosperm
host family	Pinaceae	Cistaceae	Pinaceae	Pinaceae	Pinaceae	Pinaceae	Pinaceae
host genus	*Cedrus*	*Helianthemus, Cistus*	*Cedrus*	*Cedrus*	*Pinus*	*Pinus*, *Cedrus*	*Pinus*, *Cedrus*
anti-herbivore chemicals	present	present	present	present	present	present	present
eaten foliage by larvae	mature	young	mature	mature	mature	mature	mature
**Ecology**							
foraging strategy	patch-restricted	nomadic	patch-restricted	patch-restricted	patch-restricted	central place	central place
silk tent	scarce	scarce	scarce	scarce	scarce	abundant	abundant
gregariousness as larva	present	present	present	present	present	present	present
procession	present	present	present	present	present	present	present
pupation site	soil	soil	soil	soil	soil	soil	soil
overwintering stage	egg	egg	egg	egg	egg	larva	larva
diapause	present	present	present	present	present	present	present
**Morphology**							
front of adult	crested	crested	crested	crested	crested	crested	crested
fore tibia	with spine	with spine	with spine	with spine	with spine	with spine	with spine
egg scales	present	present	present	Present	present	present	present
egg scale length mm	1.91	1.5	1.7	1.74	1.62	2.94	2.88
egg scale width mm	0.72	0.34	0.94	0.85	0.81	1.63	1.67
egg scale type	medium/medium	medium/narrow	medium/medium	medium/medium	medium/medium	long/wide	long/wide
urticating setae on larvae	present	present	present	present	present	present	present
urticating setae on moth	absent	absent	absent	absent	absent	absent	absent

Table S2. B. List of traits analysed in *Thaumetopoea* genus. Species used for the analysis (2nd part) and outgroups.

Trait	*T. processionea*	*T. solitaria*	*T. wilkinsoni*	*O. lunifer*	*H. cunea*	*L. dispar*
**Biogeography**						
distribution	widespread	localized	widespread	localized	widespread	widespread
region	Europe	East Mediterranean	East Mediterranean	Australia	North America	Europe, Asia
zoogeographic distribution	Mediterranean, Eurosibiric	Mediterranean, Iranoturanic	Mediterranean	Australian	Nearctic	Eurosibiric
**Host plant**						
host group	Angiosperm	Angiosperm	Gymnosperm	Angiosperm	Angiosperm	Angiosperm, Gymnosperm
host family	Fagaceae	Anacardiaceae	Pinaceae	Leguminosae	polyphagous	very polyphagous
host genus	*Quercus*	*Pistacia*	*Pinus*, *Cedrus*	*Acacia*	polyphagous	very polyphagous
anti-herbivore chemicals	absent	present	present	present	absent	absent
eaten foliage by larvae	young	young	mature	young	young	young
**Ecology**						
foraging strategy	central place	patch-restricted	central place	central place	nomadic	nomadic
silk tent	abundant	scarce	abundant	abundant	abundant	absent
gregariousness as larva	present	present	present	present	absent	absent
procession	present	present	present	present	absent	absent
pupation site	nest	litter	soil	litter	litter	litter
overwintering stage	egg	egg	larva	prepupa	pupa	egg
diapause	present	present	present	absent	absent	absent
**Morphology**						
front of adult	smooth	smooth	crested	smooth	smooth	smooth
fore tibia	without spine	without spine	with spine	without spine	without spine	without spine
egg scales	present	present	present	present, diverse	absent, hairs	absent, hairs
egg scale length mm	0.73	1.5	2.38	1.4		
egg scale width mm	0.15	0.47	1.59	0.2		
egg scale type	short/narrow	medium/narrow	long/wide	medium/narrow		
urticating setae on larvae	present	present	present	absent	absent	absent
urticating setae on moth	absent	absent	absent	present	absent	absent

Table S2.C. List of traits analysed in *Thaumetopoea* genus. Species not included in the analysis

Trait	*T. apologetica*	*T. cheela*	*T. dhofarensis*	*T. jordana*	*T. pseudosolitaria*	*T. sedirica*	*T. torosica*
**Biogeography**							
distribution	localized	localized	localized	localized	Localized	localized	localized
region	East Africa	India	Arabic peninsula	East Mediterranean	East Mediterranean	East Mediterranean	East Mediterranean
zoogeographic distribution	African	Iranoturanic		Saharo-arabian	Mediterranean, Iranoturanic	Mediterranean	Mediterranean
**Host plant**							
host group	*	Angiosperm	*	Angiosperm	Angiosperm	Gymnosperm	Gymnosperm
host family	*	Anacardiaceae	*	Anacardiaceae	Fagaceae	Pinaceae	Pinaceae
host genus	*	*Rhus cotinus*	*	*Rhus tripartita*	*Quercus*	Pinus	*Pinus*
anti-herbivore chemicals	*	*	*	present	absent	present	present
eaten foliage by larvae	*	*	*	young	young	*	*
**Ecology**							
foraging strategy	*	*	*	*	*	*	*
silk tent	*	*	*	scarce	*	*	*
gregariousness as larva	*	*	*	present	present	*	*
procession	*	*	*	present	present	*	*
pupation site	*	*	*	*	*	*	*
overwintering stage	*	*	*	larva	egg	*	*
diapause	*	*	*	present	*	*	*
**Morphology**							
front of adult	crested	crested	crested	crested	smooth	*	*
fore tibia	*	*	*	with spine	without spine	*	*
egg scales	*	*	*	present	*	*	*
egg scale length mm	*	*	*	3	*	*	*
egg scale width mm	*	*	*	0,48	*	*	*
egg scale type	*	*	*	long/narrow	*	*	*
urticating setae on larvae	*	*	*	present	present	*	*
urticating setae on moth	*	*	*	absent	absent	*	*

*, not available

List of References consulted to produce the Table S2.

Agenjo R. 1941. Monografia de la familia Thaumetopoeidae (Lep.). *Eos* **17**:69-130.

Androic M. 1956. Contribution à l'étude de *Cnethocampa pityocampa* Schiff. *Revue de Pathologie Végetale et d'Entomologie Agricole de France* **35**:251-262.

Avtzis N. 1986. Development of Thaumetopoea pityocampa schiff. (Lepidoptera: Thaumetopoeidae) in relation to food consumption. *Forest Ecology and Management* **15**:65-68.

Avtzis N. 1997. Occurrence of the pine processionary (*Thaumetopoea pityocampa* Schiff., Lep.: Thaumetopoeidae) in Greece. *Mitteilungen der Deutschen Gesellschaft fur Allgemeine und Angewandte Entomologie* **11**:699-703.

Buxton RD. 1990. The influence of host tree species on timing of pupation of *Thaumetopoea pityocampa* Schiff. (Lep., Thaumetopoeidae) and its exposure to parasitism by *Phryxe caudata* Rond. (Dipt., Larvaevoridae) *Journal of Applied Entomology* **109**:302-310.

Ciesla WM. 2004. Forests and forest protection in Cyprus. *Forestry Chronicle* **80**:107-113.

de Freina J,Witt JT. 1982. Taxonomische Veranderungen bei den Bombyces und Sphinges Europas und Nordwestafrikas (Lepidoptera: Thaumetopoeidae, Ctenuchidae). *Atalanta* **13**:309-317.

de FreinaJ, Witt TJ. 1987. Die Bombyces und Sphinges der Westpalaerktis (Insecta, Lepidoptera) 1. Forschung und Wissenschaft, München. 708 pages, 46 col plates, 330 maps.

Demolin G. 1988. Intensification de la protection phytosanitaire des forets. Algerie 1986-1987. La processionnaire du cedre: *Thaumetopoea bonjeani*. FAO - Rapport scientifique et rapport iconographique, Rome.

Demolin G, Frerot B, Chambon JP, Martin E. 1994. Reflexion biosystematiques sur toutes le processionnaires du genre *Thaumetopoea* (Lep. Thaumetopoeidae), considérées comme ravageurs importants des cèdres *Cedrus libani* Barel et *Cedrus atlantica* Manetti sur le pourtour du bassin mediterraneen. *Annales des Recherches Forestières Maroc* **2**:577-591.

Demolin G, Nemer N, Villemant C. 1999. Defoliator insects of *Quercus callyprinos* Webb. and *Quercus infectoria* Oliv. in Lebanon. *Bulletin OILB/SROP* **22**:65-69.

Devkota B, Schmidt GH. 1990. Larval development of *Thaumetopoea pityocampa* (Den and Schiff) (Lep, Thaumetopoeidae) from Greece as influenced by different host plants under laboratory conditions. *Journal of Applied Entomology-Zeitschrift Fur Angewandte Entomologie* **109**:321-330.

Doganlar M, Avci M. 2001. A new species of *Traumatocampa* Wallengren (Lepidoptera: Thaumetopoeidae) feeding on cedar from Isparta (Turkiye). *Turkiye Entomoloji Dergisi* **25**:19-22.

Doganlar M, Doganlar O, Doganlar F. 2005. Morphology and systematics of European species of *Traumatocampa* Wallengren, 1871 with descriptions of two new species from the Mediterranean region of Turkey (Lepidoptera, Thaumetopoeidae). *Entomofauna Zeitschrift fur Entomologie* **26**:229-240.

El Yousfi M. 1989. The cedar processionary moth, *Thaumetopoea bonjeani* (Powell). Boletin de Sanidad Vegetal, Plagas **15**:43-56.

Frerot B, Demolin G. 1993. Sex pheromone of processionary moths and biosystematic considerations within the genus *Thaumetopoea* (Thaumetopoeidae Thaumetopoeinae). *Bollettino di Zoologia Agraria e di Bachicoltura* **25**:33-40.

Furth DG, Halperin J. 1979. Observations on the phenology and biogeography of *Thaumetopoea jordana* (Staudinger) (Lepidoptera Thaumetopoeidae). *Israel Journal of Entomology* **13**:1-11.

Gabler H. 1954. Prozessionspinnner. Ziemsen, Wittenburg.

Gomez-Bustillo MR, Bustillo MG. 1978. The Thaumetopoeidae (Aurivillius, 1891) of the Iberian peninsula: notes on systematics, ecology and the economic importance of the family. (Second part). *Revista de Lepidopterologia, SHILAP* **6**:113-124.

Grison P, de Sacy SR, Galichet PF. 1951. La processionnaire du pin (*Thaumetopoea pityocampa* Schiff.). *Revue de Zoologie Agricole et Appliquee* **50**:1-33.

Halperin J. 1990. Life history of *Thaumetopoea* spp. (Lep., Thaumetopoeidae) in Israel. *Journal of Applied Entomology* **110**:1-6.

Halperin J, Sauter W. 1999. The occurrence of *Thaumetopoea processionea* L. (Lep.: Thaumetopoeidae) on Mt. Hermon. *Phytoparasitica* **27**:107.

Hódar J.A, Zamora R. 2004. Herbivory and climatic warming: a Mediterranean outbreaking caterpillar attacks a relict, boreal pine species. *Biodiversity and Conservation* **13**:493-500.

Hódar JA, Zamora R, Castro J. 2002. Host utilisation by moth and larval survival of pine processionary caterpillar *Thaumetopoea pityocampa* in relation to food quality in three *Pinus* species. *Ecological Entomology* **27**:292-301.

Huchon H, Demolin G. 1971. The bioecology of the Pine processionary [*Thaumetopoea pityocampa*]. Potential and current distribution. *Phytoma* **23**:11-20.

Kerdelhué C, Zane L, Simonato M, Salvato P, Rousselet ., Roques A, Battisti A. 2009. Quaternary history and contemporary patterns in a currently expanding species. *BMC Evolutionary Biology* **9**.

Kiriakoff SG. 1970. Lepidoptera Familia Thaumetopoeidae.*in* P. Wytsman, editor. Genera Insectorum. SPRL, Anvers Belgium.

Kiriakoff SG, Talhouk AS. 1975. *Thaumetopoea libanotica* spec.nov. *Opuscula Zoologica* **137**:1-5.

Koch, M. 1953. Zur Biologie des Kiefernprozessionsspinners, *Thaumetopoea pinivora* Tr. . *Beitrage Entomologie* **4**:423-427.

Lamy M, Novak F. 1987. The oak processionary caterpillar (*Thaumetopoea processionea* L.) an urticating caterpillar related to the pine processionary caterpillar (*Thaumetopoea pityocampa* Schiff.) (Lepidoptera, Thaumetopoeidae). *Experientia* **43**:456-458.

Mendel Z. 1987. Major pests of man-made forests in israel: origin, biology, damage and control. *Phytoparasitica* **15**:131-137.

Mendel Z. 1988. Host selection by the pine processionary caterpillar *Thaumetopoea wilkinsoni*. *Phytoparasitica* **16**:101-108.

Paiva MR, Mateus E, Santos MH, Branco MR. 2011. Pine volatiles mediate host selection for oviposition by *Thaumetopoea pityocampa* (Lep., Notodontidae). *Journal of Applied Entomology* **135**:195-203.

Palanca SA, Castan LC, Calle PJ, Soler AP, Lanaspa CC, Pascual JC. 1982. *Thaumetopoea galaica*, a species new to science (preliminary note). *Revista de Lepidopterologia, SHILAP* **38**:92.

Pérez-Contreras T, Soler JJ, Soler M. 2003. Why do pine processionary caterpillars *Thaumetopoea pityocampa* (Lepidoptera, Thaumetopoeidae) live in large groups? An experimental study. *Annales Zoologici Fennici* **40**:505-515.

Petrakis PV, Roussis V, Papadimitriou D, Vagias C, Tsitsimpikou C. 2005. The effect of terpenoid extracts from 15 pine species on the feeding behavioural sequence of the late instars of the pine processionary caterpillar *Thaumetopoea pityocampa*. *Behavioural Processes* **69**:303-322.

Pimentel C, Calvão T, Ayres MP. 2011. Impact of climatic variation on populations of pine processionary moth *Thaumetopoea pityocampa* in a core area of its distribution. *Agricultural and Forest Entomology* **13**:273-281.

Ronnås C, Larsson S, Pitacco A, Battisti A. 2010. Effects of colony size on larval performance in a processionary moth. *Ecological Entomology* **35**:436-445.

Rousselet, J., R. Zhao, D. Argal, M. Simonato, A. Battisti, A. Roques, and C. Kerdelhué. 2010. The role of topography in structuring the demographic history of the pine processionary moth, *Thaumetopoea pityocampa* (Lepidoptera: Notodontidae). *Journal of Biogeography* **37**:1478-1490.

Santos H, Burban C, Rousselet J, Rossi JP, Branco M, Kerdelhué C. 2011. Incipient allochronic speciation in the pine processionary moth (*Thaumetopoea pityocampa*, Lepidoptera, Notodontidae). *Journal of Evolutionary Biology* **24**:146-158.

Schmidt GH. 1990. On the biology and control of *Thaumetopoea* spp. (Lepidoptera,Insecta). Proceedings of the *Thaumetopoea*-Symposium 5-7 July 1989 at Neustadt a.Rbge near Hannover.

Schopf R, Avtzis N. 1987. The importance of needle compounds on the predisposition of 5 pine species to the attack by *Thaumetopoea pityocampa* (Schiff.). *Journal of Applied Entomology-Zeitschrift Fur Angewandte Entomologie* **103**:340-350.

Simonato M, Mendel Z, Kerdelhué C, Rousselet J, Magnoux E, Salvato P, Roques A, Battisti A, Zane L. 2007. Phylogeography of the pine processionary moth *Thaumetopoea wilkinsoni* in the Near East. *Molecular Ecology* **16**:2273-2283.

Tams WHT. 1925. A new processionary month (Notodontidae) injurious to pine trees in cyprus. *Bulletin of entomological research* **15**:293-294.

Wilkinson DS. 1926. The Cyprus processionary caterpillar (*Thaumetopoea wilkinsoni*, Tams). *Bulletin of entomological research* **18**:163-182.
